# A Convenient Synthesis of Short α-/β-Mixed Peptides as Potential α-Amylase Inhibitors

**DOI:** 10.3390/molecules29174028

**Published:** 2024-08-26

**Authors:** Naeem Ahmed, Fakhira Razzaq, Muhammad Arfan, Mansour K. Gatasheh, Hammad Nasir, Joham Sarfraz Ali, Hamna Hafeez

**Affiliations:** 1 Department of Chemistry, School of Natural Sciences, National University of Sciences and Technology, Islamabad 44000, Pakistan; naeem.ahmed@sns.nust.edu.pk (N.A.); frazzaq@mtu.edu (F.R.); hammadn530@gmail.com (H.N.); hhamna497@gmail.com (H.H.); 2 Department of Chemistry, Michigan Technological University, 1400 Townsend Dr., Houghton, MI 49931, USA; 3 Department of Biochemistry, College of Science, King Saud University, P.O. Box 2455, Riyadh 11451, Saudi Arabia; mgatasheh@ksu.edu.sa; 4 The Department of Biological Sciences (DBS), National University of Medical Sciences (NUMS), Rawalpindi 46000, Pakistan; joham.sarfraz@numspak.edu.pk

**Keywords:** α-/β-mixed peptides, β-amino acid, postprandial hyperglycemia, Arndt–Eistert synthesis

## Abstract

Over the last decades, the increased incidence of metabolic disorders, such as type two diabetes and obesity, has motivated researchers to investigate new enzyme inhibitors. Inhibition of the α-amylase enzyme is one therapeutic approach in lowering glucose levels in the blood to manage diabetes mellitus. The objective of this study was to synthesize short α-/β-mixed peptides in the solution phase. The Boc-protected α-L-leucine was converted to β-analogue by using Arndt–Eistert synthesis with the advantage of no racemization and retention of configuration. Three novel short peptides were successfully synthesized: N(Boc)-Gly-β-Leu–OCH_3_(14), N(Boc)-O(Bz)α-Ser-β-Leu–OCH_3_(16), and N(Boc)-O(Bz)-α-Tyr-α-Gly-β-Leu–OCH_3_(17), characterized by FTIR and ^1^H NMR analysis. The synthesized peptide 16 showed highest inhibitory activity (45.22%) followed by peptide 14 (18.51%) and peptide 17 (17.05%), respectively. Intriguingly, peptide 16 showed higher inhibition on α-amylase compared with other α-/β-mixed peptides.

## 1. Introduction

The World Health Organization (WHO) reported that the global count for diabetes was 463 million in 2019, expected to reach 700 million in 2045. Diabetes mellitus is a metabolic disease resulting in chronic hyperglycemia caused by problems with insulin secretion, insulin action, or both. If taken not seriously, it may cause numbness, coma, and death due to ketoacidosis [1,2]. Typically, 90–95% of reported cases are due to Type-2 diabetes, estimated to reach 366 million in 2030 [3]. A patient with Type-2 diabetes may suffer severe changes in the blood vessels, nerves, and immune system [4,5]. Therefore, diabetes requires more attention for suitable treatments for short-term complications like hyperglycemia, eyesight problems, and fatigue and long-term issues such as cardiovascular disease, renal failure, and neuropathy [5].

It is observed that postprandial hyperglycemia management after a meal is the most favorable remedy for diabetes, as postprandial hyperglycemia becomes remarkably intense when glucose absorption occurs in the intestine [6]. The α-amylase is a critical enzyme in saliva and pancreatic juice, and it is considered a favorable target for regulating postprandial hyperglycemia [7]. Contrary to α-glucosidases, which act in the last stage of starch hydrolysis, α-amylase hydrolyzes giant starch molecules into small absorbable molecules [8]. It is, therefore, productive to suppress the action of α-amylase as a treatment choice for diabetes patients [9]. So, inhibitors can hamper the absorption of carbohydrates, decline postprandial hyperglycemia, and possibly be helpful for diabetes patients [10].

Many drugs for the treatment of type-2 diabetes have entered the market in the last two decades, but their side effects have been identified. Metformin is the first agent used as monotherapy, with gastrointestinal upset as the significant side effect [11]. As a monotherapy, sulphonylureas are also employed, but they cause weight gain and hypoglycemia [12]. Similarly, Glinides show respiratory infections and drug interactions which results in hypoglycemia and weight gain [13]. On the other hand, using thiazolidinediones causes weight gain, edema, bone loss, and heart failure [14,15,16]. Moreover, Dipeptidyl peptidase-4 inhibitors show undesirable side effects of angioedema and pancreatitis [17,18].

**Figure 1:**
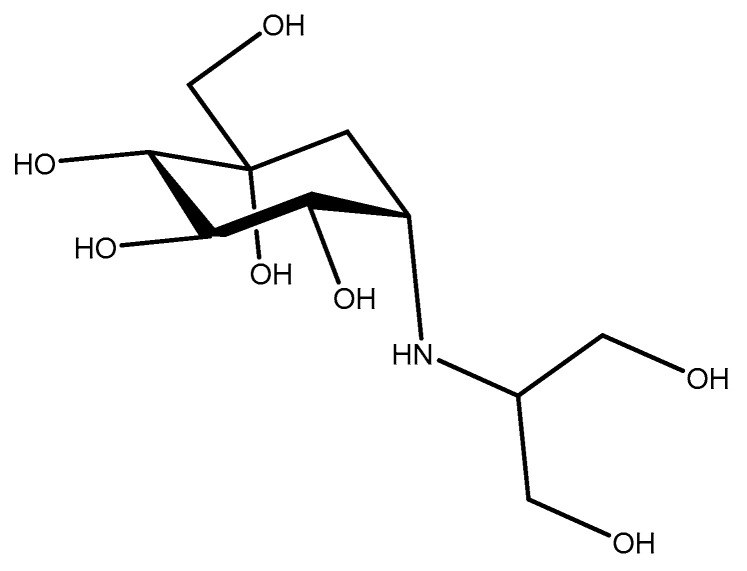
Voglibose.

**Figure 2:**
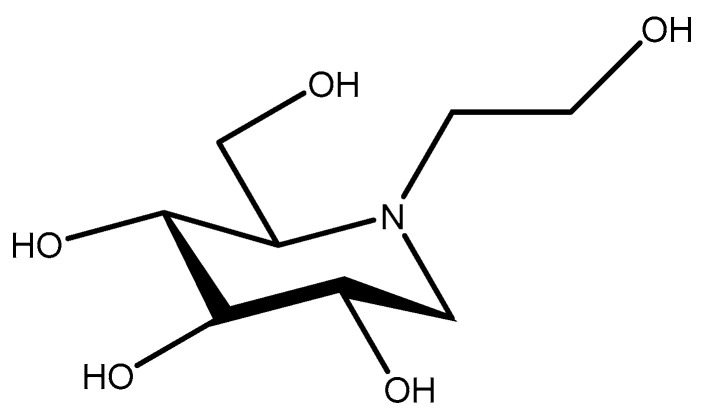
Miglitol.

**Figure 3:**
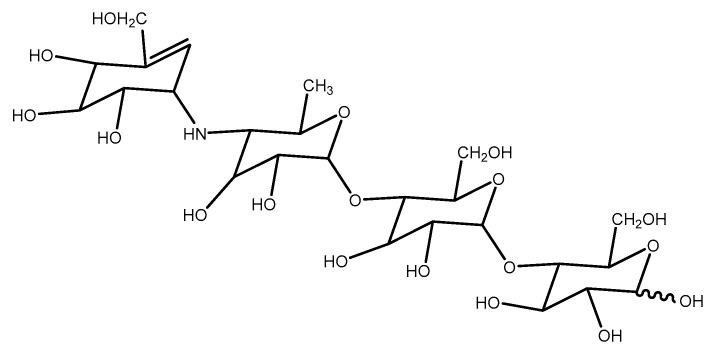
Acarbose.

The drugs Voglibose (Figure 1), Miglitol, (Figure 2), and Acarbose (Figure 3) are currently used in clinics. Miglitol and Voglibose act on α-glucosidases. They effectively control blood glucose levels after meals but are associated with many side effects [16,17,18,19]. Acarbose inhibits both α-amylase and brush border α-glucosidases [20]. Unfortunately, acarbose also shows unwanted side effects and, in limited cases, is linked to hepatotoxicity [21]. Therefore, there is an urgent need to develop molecules that can inhibit carbohydrate metabolism without side effects and control postprandial glucose levels in the blood.

**Figure 4:**
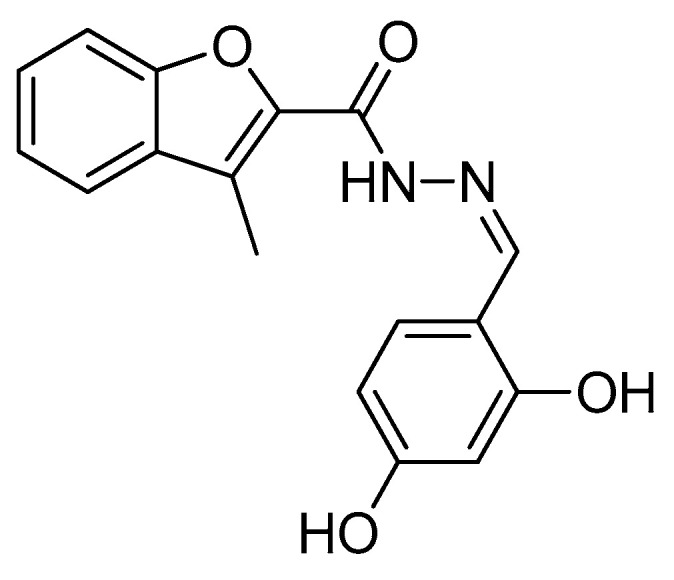
Benzofuran hydrazone.

**Figure 5:**
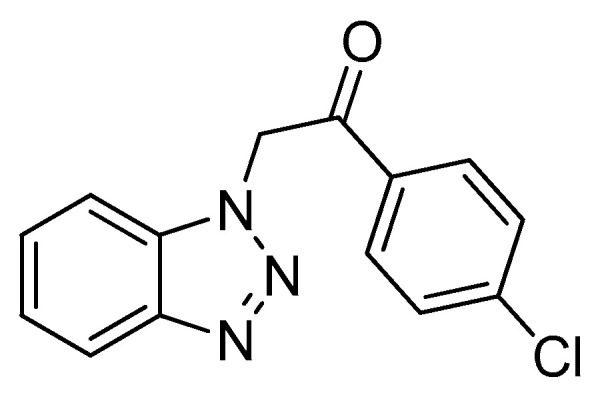
Benzotriazole.

**Figure 6:**
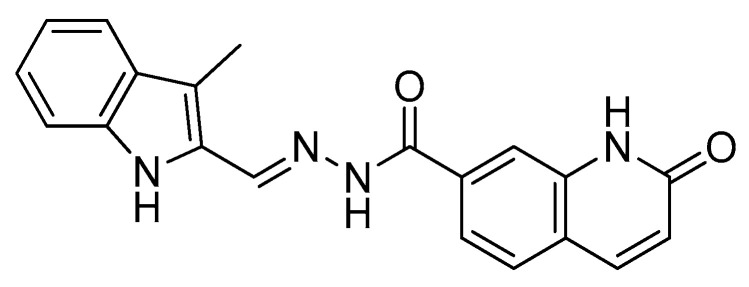
Indole hydrazone.

Much research on the inhibitory action of several compounds against α-amylase enzymes has been reported. The derivatives of drug molecules such as benzofuran hydrazone (Figure 4) [22], benzotriazoles (Figure 5) [23], and indole hydrazone (Figure 6) [24] have been developed.

Bioactive peptides are small parts of parent proteins that are healthy for body functions and may impact human lives [25]. They are inactive in parent protein and released mainly by enzymatic hydrolysis. Bioactive peptides with α-amylase inhibitory peptides have been isolated from various sources such as cumin seeds, pinto bean, royal jelly, common bean, and egg-white albumin [8,9,26,27,28]. A significant challenge with using peptides as drugs is their vulnerability to enzymatic hydrolysis when administered in vivo. To address this issue, various chemical strategies have been explored, including the incorporation of D-amino acids, unnatural amino acids, α,α-disubstituted amino acids, cyclic moieties, and cyclization of peptides [29,30,31]. One approach involves substituting α-amino acids with β-amino acids, resulting in β-peptides.

β-peptides are oligomers of β-amino acids [32,33], offering greater structural diversity due to the presence of an additional carbon atom in each amino acid residue. These β-peptides exhibit distinct dimensions, geometry, and polarities compared to α-peptides, leading to differences in their biological properties. Notably, β-peptides do not bind to the active sites of human peptidases, making them entirely stable against proteolytic degradation [34,35]. Despite these differences, β-peptides can still mimic the pharmacological activity of α-peptides, especially small β-peptides with strong folding preferences.

**Figure 7:**
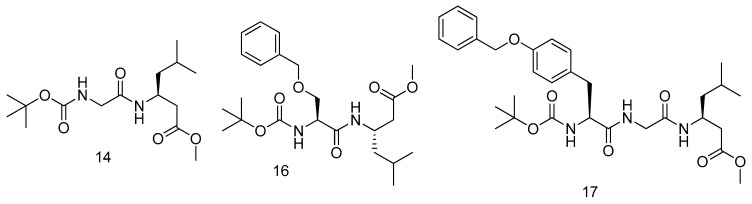
Structures of synthesized short α-/β-mixed peptides.

Previous reports have demonstrated that, in short α-peptides, the binding of α-amylase is strongly affected by the amino acids Leu, Ser Gly, and Tyr. Especially inspired by walnut-derived (*Juglans mandshurica Maxim.*) [36] α-peptide LPLLR and red seaweed (*Porphyra species*) [37]-derived LS and GGSY having a high percentage of Leu, Ser, and Gly, we synthesized short α-/β-mixed peptides through the solution phase, incorporating Ser, Gly, and Tyr as α-amino acids and Leu as β-amino acid. Boc-protected α-Leucine was converted to the respective β-methyl-ester derivative by the Arndt–Eistert method [38]. The advantage and significance of this method is no racemization. After Boc deprotection, [39], TFA salts of β-methyl-esters were coupled [40] with *N*-protected α-amino acids to give short α-/β-mixed peptides (Figure 7), and their α-amylase activity is reported by our research group for the first time.

## 2. Results and Discussion

### 2.1. Chemistry

Synthesized compounds were characterized by spectral data (IR and ^1^H NMR). For compounds, each proton signal in nuclear magnetic resonance (^1^H NMR, δ ppm) was confirmed based on their chemical shifts, multiplicities, and coupling constants.

**Scheme 1:**
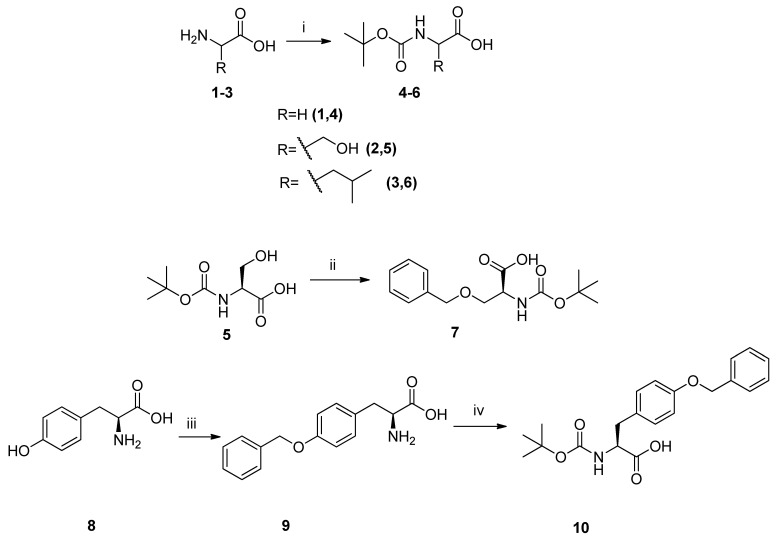
(i) (Boc)_2_O, (Et)_3_N, (1:1) Dioxane/Water, 8 h; (ii) Benzyl Bromide, NaH, DMF; (iii) 2M NaOH, CuSO_4_.5H_2_O, Benzyl Bromide; (iv) (Boc)_2_O,1N NaOH, NaHCO_3_, (2:1) Dioxane/Water.

*N*-Boc-protected amino acids of our desired sequence, like Glycine **1**, L-Serine **2,** and L-Leucine **3,** were treated with (Boc)_2_O to obtain *N*-Boc-glycine **4**, *N*-Boc-L-serine **5,** and *N*-Boc-L-leucine **6**, respectively. FTIR spectra of compounds **4**–**6** protected with Boc were indicated by IR bands 1613–1673 cm^−1^ for (C=O carbamate) and 1365–1380 cm^−1^ (C(CH_3_)_3_) for tertiary butyl from the Boc-protecting group (Scheme 1). Compound *N*-Boc-*O*-benzyl-L-serine **7** was obtained via Williamson synthesis [32] by reaction of *N*-Boc-serine **5** with benzyl bromide. IR bands appearing at 1157 cm^−1^ (C–O ether) in compound **7** indicated that side chain OH protection was performed successfully with the benzyl group. L-Tyrosine **8** was converted to *O*-benzyl-L-tyrosine **9** by benzylation through copper complex formation under alkaline pH [41]; IR bands at 1117 (C–O ether) and 874 cm^−1^ (para-substitution) indicated our desired compound formation. Using (Boc)_2_O as a protecting group in basic reaction conditions, compound *O*-Benzyl-L-tyrosine **9** was converted to *N*-Boc-*O*-benzyl-L-tyrosine **10** (Scheme 1).

**Scheme 2:**
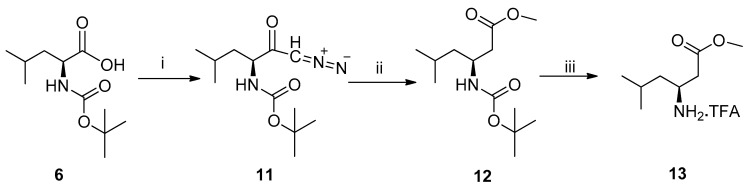
(i) Ethyl chloroformate, Et_3_N, THF, N_2_, −15 to −5 °C, CH_2_N_2_ in ether; (ii) Silver Benzoate, Et_3_N, dark, −25 °C, dry MeOH; (iii) TFA dry DCM, 0 °C.

Compound *N*-Boc-L-leucine **6** was activated with ethyl chloroformate in the presence of triethylamine and converted to *N*-Boc-L-leucine Diazoketone **11** by treatment with freshly generated diazomethane [38], and its synthesis was confirmed by the presence of a singlet at δ 6.1 ppm (–CH=N_2_) integrated for one proton (Figure S8). Compound **11** was converted to its β-methyl ester **12** via wolf rearrangement [42], in the presence of silver benzoate as a catalyst in dry methanol under dark conditions. In the ^1^H NMR spectrum of compound **12**, a singlet at δ 3.62 (–OCH_3_) integrated for three protons confirmed the formation of the desired β-methyl ester (Figure S11). Compound *N*-Boc-L-leucine-β-methyl ester **12** was Boc-deprotected by using TFA in dry DCM to give **13**, and its deprotection was indicated by the absence of an IR band for the Boc group in the IR spectrum (Scheme 2).

For peptide synthesis, Boc-deprotected compound **13** was coupled with Boc-protected *N*-Boc-α-glycine compound **4** in the presence of coupling agent EDC and HOBt to give compound **14** as dipeptide, which was confirmed by the presence of broad singlet at δ 6.85 ppm (–NH) integrated for one proton and the presence of a Boc peak as singlet at δ 1.41 ppm integrated for nine protons (Figure S16). The appearance of a peak in the ^13^C spectrum at 170.59 ppm confirms that the amide bond is successfully formed, and the peak at 155.84 ppm confirms that the Boc protection is intact (Figure S18). Similarly, Boc-protected *N*-Boc-*O*-benzyl-L-serine **7** and Boc-deprotected compound **13** were coupled to yield α-/β-dipeptide, i.e., *N*-Boc-*O*-benzyl-α-serine-β-leucine-methyl ester **16**. The IR bands at 1615 for (C=O amide) group and 1731(C=O ester) group indicated the formation of our desired peptides. In the ^1^H NMR spectrum, a multiplet at 7.30–7.25 ppm, integrating for five protons, confirms the presence of the benzyl group (Figure S20). A singlet at 3.56 ppm, integrating for three protons, is characteristic of the methyl ester. Additionally, a nine-proton singlet at 1.40 ppm is consistent with the tert-butyl group of the Boc-protecting group. The ^13^C NMR spectrum further confirms the structure. Signals at 172.83 and 171.02 ppm are consistent with the two carbonyl groups in the molecule (ester and amide). Furthermore, the signal at 156.12 ppm is characteristic of the carbonyl group within the Boc-protecting group (Figure S21).

**Scheme 3:**
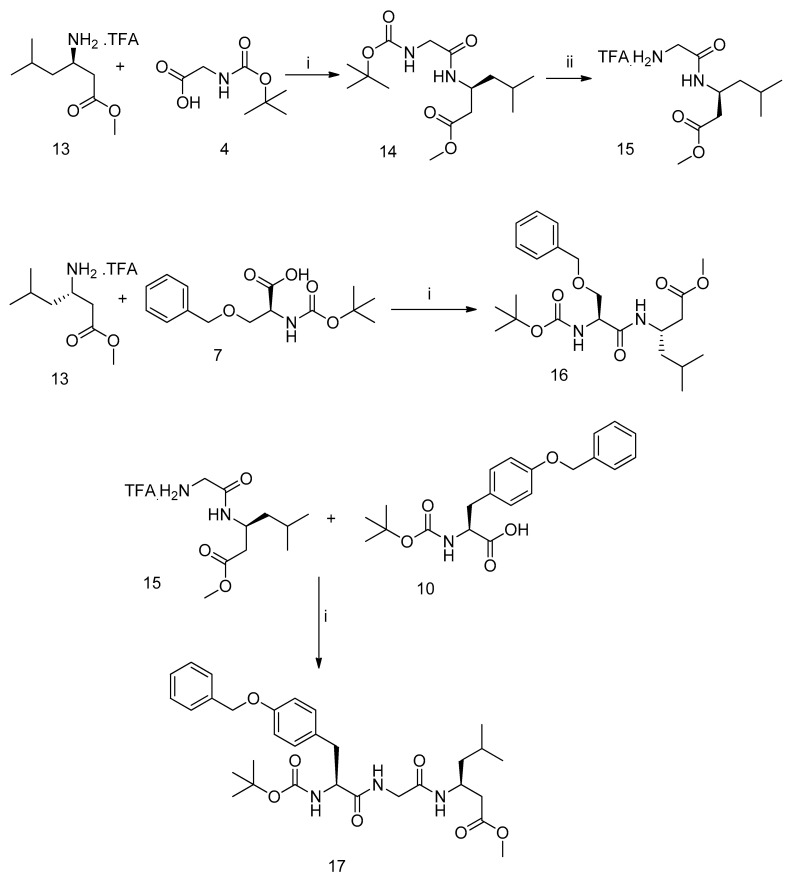
Synthesis of α-/β-mixed peptides: (i) EDC, HOBt, Et_3_N, 0 °C, CHCl_3_, N_2_, (ii) TFA, Dry CH_2_Cl_2_, N_2_ atmosphere.

TFA was used to remove the Boc group of compound **14** in dry DCM to yield Boc-deprotected dipeptide **15** as TFA salt [29]. The absence of the IR band of the (CH_3_)_3_C group at 1365 cm^−1^ in IR spectrum of compound 15 indicated its deprotection successfully. Then, compound **15** as TFA salt of dipeptide and Boc benzyl-protected tyrosine (**10**) were coupled [30] in the presence of EDC and HOBt to afford compound **17** as tripeptide. A singlet at δ 1.50 ppm integrated for nine protons for the Boc group and another singlet at δ 3.65 for –OCH_3_ integrated for three protons in the ^1^H NMR spectrum confirmed the synthesis of our desired α-/β-mixed peptide **17** (Figure S24). In the ^13^C spectrum, two distinct peaks at 171.80 ppm and 170.27 ppm in the carbonyl region strongly suggest the presence of two amide bonds. The peak at δ 155.61 ppm indicates carbonyl attached to hetero atom characteristics of Boc protection, confirming that the Boc is still attached (Figure S26). These data also confirm compound **17** (Scheme 3).

### 2.2. α-Amylase Inhibition Potential

α-Amylase contributes to a variety of disease disorders through normal metabolic processes. α-Amylase inhibitors function by inhibiting the enzyme, alleviating related disorders’ symptoms [43,44]. The ability of plant-based products to suppress α-amylase has already been described [45]. A peptide with α-amylase inhibitory capacity is a superior choice for antidiabetic-management-targeted research [46,47,48]. In this study, we have synthesized three novel short α-/β-mixed peptides and reported in vitro α-amylase inhibitory activity for the first time.

The novel synthesized peptides of this study are N(Boc)-O(Bz)-α-Ser-β-Leu–OCH_3_ **16**, a tripeptide, and two dipeptides, N(Boc)-O(Bz)-α-Tyr-α-Gly-β-Leu–OCH_3_ **17** and N(Boc)-Gly-β-Leu–OCH_3_ **14**. The results of the α-amylase inhibitory activity suggested that these peptides show low-to-moderate potential against the target enzyme (Table 1). The dipeptide N(Boc)-O(Bz)-α-Ser-β-Leu–OCH_3_ **16** showed the highest α-amylase inhibition. The other two N(Boc)-O(Bz)-α-Tyr-α-Gly-β-Leu–OCH_3_ **17** and N(Boc)-Gly-β-Leu–OCH_3_ **14** exhibited almost the same α-amylase inhibitory potential. The peptide with moderate activity N(Boc)-O(Bz)-α-Ser-β-Leu–OCH_3_ **16** was almost 27% more active than both N(Boc)-O(Bz)-α-Tyr-α-Gly-β-Leu–OCH_3_ **17** and N(Boc)-Gly-β-Leu–OCH_3_ **14**. However, information from the literature is limited for the α-amylase potential of α-/β-mixed short peptides. According to our knowledge, this is the first study regarding α-/β-mixed short peptides as α-amylase inhibitors. However, some studies have been reported on the α-amylase inhibition of short α-peptides isolated from natural sources, e.g., peptides with amino acid sequence Lys-Leu-Pro-Gly-Phe from peptide hydrolysate of albumin [47], Leu-Gly-Gly-Gly-Asn from protein hydrolysate of the Chinese giant salamander (*Andriasdavidianus*) [48], Leu-Pro-Leu-Leu-Arg identified from walnut protein hydrolysates [36], and Gly-Gly-Ser-Leu and Glu-Leu-Ser from protein hydrolysates of red seaweed laver (*Porphyra* species) [37].

Siow et al. (2015) synthesized three peptides, CSP1(FFRSKLLSDGAAAAKGALLPQYW), CSP2(RCMAFLLSDGAAAAQQLLPQYW), and CSP3 (DPAQPNYPWTAVLVFRH) derived from cumin seeds [49]. Peptide CSP1 showed α-amylase inhibition of 24.54%, and these peptides are large (17 to 23 mer) compared to our synthesized short peptides (2–3 Mer). Moreover, their three peptides showed less α-amylase inhibition than our synthesized α-/β-mixed peptides. The short peptides also offer advantages of bioavailability, specificity, rapid action, cell permeability, and ease of synthesis as compared to large peptides. The synthesized peptide of this work, N(Boc)-O(Bz)-α-Ser-β-Leu–OCH_3_ **16**, was 20% more potent than CSP1. But N(Boc)-Gly-β-Leu–OCH_3_ (**14**) and N(Boc)-O(Bz)-α-Tyr-α-Gly-β-Leu–OCH_3_ (**17**) exhibited lower inhibition of 18.51% and 17.05%, respectively. However, the α-amylase inhibition can be improved by further modification of the amino acid sequence in the synthesized α-/β-peptides. Some protein hydrolysates have been reported as amylase inhibitors. For instance, Yu et al. (2011) reported peptides from egg-white protein which exhibited no detectable α-amylase inhibition [47]. Hence, the short α-/β-mixed peptides of this investigation potentially contribute as α-amylase inhibitors and could be modified as potential antidiabetic agents.

It has been reported that amino acid composition and sequence may affect α-amylase activity, and L-Leucine and L-Serine are found in previously reported α-peptides [36,37,47,48]. The presence of Leucine and Serine in peptide is linked with their binding affinity towards α-amylase enzymes, making these peptides a potent source of inhibitors, facilitating their crucial role as antidiabetic agents [50]. Leucine is thought to be important in increasing insulin production by membrane depolarization and allosteric stimulation of metabolism [51]. The short α-peptides Glu-Leu-Ser and Gly-Gly-Ser-Leu, which showed α-amylase inhibition, were identified from red seaweed laver (*Porphyra species*) by enzymatic hydrolysis and have L-Serine and L-Leucine residues [37]. The short α-peptide Leu-Pro-Leu-Leu-Arg, identified from walnut (Juglans mandshurica Maxim), exhibited 39.08% α-amylase activity [36]. One of our synthesized peptides in this research work, N(Boc)-O(Bz)-α-Ser-β-Leu–OCH_3_ **16**, having α-L-Serine and β-L-Leucine in sequence, exhibits 45% α-amylase activity, more than the α-peptide Leu-Pro-Leu-Leu-Arg from walnut [36]. The synthesized short α-/β-mixed peptides have shown low-to-moderate inhibitory effects for α-amylase. This study suggests that further structural modification and changing the sequence of α- and β-amino acids in these peptides may further increase the α-amylase activity and may be used as lead compounds in the future. Moreover, the presence of β-amino acid in synthesized peptides may be beneficial as compared to natural short α-peptides in terms of proteolytic degradation both in vitro and in vivo [52,53,54].

Collectively, the mechanisms by which protein hydrolysates, bioactive peptides, and amino acids control glucose levels are still ambiguous, relating it to a series of complex pathways of regulation and inhibition of glucose within the peripheral tissues. This demands a detailed and extensive study of its mechanisms and kinetics to better understand the mode of action of peptides [44].

In conclusion, we reported the synthesis and characterization of short α-/β-mixed peptides N(Boc)-O(Bz)-α-Ser-β-Leu–OCH_3_ **16**, N(Boc)-O(Bz)-α-Tyr-α-Gly-β-Leu–OCH_3_ **17**, and N(Boc)-Gly-β-Leu–OCH_3_ **14**. These novel short α-/β-mixed peptides have shown α-amylase inhibitory activity which, in most cases, is better than isolated and synthesized reported peptides. Interestingly, the peptide N(Boc)-O(Bz)-α-Ser-β-Leu–OCH_3_ **16** presented the highest α-amylase inhibitory action, which may further be improved by modification of sequence and structure of these peptides by using different α- and β-amino acids. Furthermore, these compounds may act as lead compounds for antidiabetic therapeutics with proteolytic stability.

## 3. Materials and Methods

Chemicals/reagents were purchased from Merck/Sigma-Aldrich (Bremen, Germany). A mini diazald apparatus was purchased from Sigma-Aldrich with clear-seal joints. Staffordshire, ST15-OSA, UK capillary melting point apparatus was used for melting points. Reaction progress was monitored by thin-layer chromatography and visualized under a UV lamp. Different stains, ninhydrin, iodine vapors, and KMnO_4_ solution were used as locating agents. Optical rotations were obtained from ATAGO, an automated polarimeter, with concentrations in g/100 mL. ^1^H NMR spectra were recorded on a Bruker AC-300 NMR (300 MHz) instrument. Chemical shifts and coupling constants were given in Hz and ppm. Infrared (IR) spectra were taken on a Bruker-Alpha Platinum, ATR (Bremen, Germany), and elemental analysis was performed on a CHNS Analyzer, CKTC-SECHN200.

### 3.1. Synthesis of Protected Amino Acids

#### 3.1.1. Synthesis of Boc-Protected Amino Acid (**4**–**6**)

Glycine (**1**) (0.75 g, 10 mmol), L-Serine (**2**) (1.05 g, 10 mmol), and L-Leucine (**3**) (1.31 g, 10 mmol) were dissolved in a mixture of dioxane and water (1:1, 42 mL). The Et_3_N (1.6 eq, 2.2 mL) was added, then followed by (Boc)_2_O (2.4 g, 11 mmol) at 0 °C. The reaction was allowed to stir for 8 h at room temperature. After rotary evaporation, distilled water was added, and the pH was adjusted to 1–2 using KHSO_4_ solution. After extraction with ethyl acetate, combined organic layers were washed with NaCl solution, dried over anhydrous MgSO_4_, filtered, and concentrated using a rotary evaporator. The crude products were purified by column chromatography using an ethyl acetate/n-hexane solvent system, resulting in pure products with yields 91–93% [40].

*N*-Boc-Glycine (**4**). Reaction time: 18 h, yield: 93%, physical appearance: white powder, M.P: 86–89 °C, TLC system: (30% ethyl acetate/n-hexane, Stain, Ninhydrin), [α]_D_^25^: +0.04° (c = 5 mg/10 mL CH_3_OH), FTIR (neat, v_max_): 2976 (C–H, CH_2,_ str), 1737 (C=O, acid), 1665 (C=O, carbamate), 1408 (C–N, carbamate), 1367 (C(CH_3_)_3_, 1195 (C–O) cm^−1^. Anal. calcd. for C_7_H_13_NO_4_ (175.18): C, 47.89; H, 7.42; N, 8.00. Found: C, 47.86; H, 7.40; N, 8.00.

*N*-Boc-Serine (**5**). Reaction time: 24 h, yield: 92%, physical appearance: colorless thick gel, TLC system: (20% methanol/chloroform, Stain, Ninhydrin), [α]_D_^25^: −0.21 (c = 3 mg/10 mL, CH_3_OH), FTIR (neat, v_max_): 2979 [C–H, CH_2,_ str], 1710 (C=O, acid), 1689 (C=O, carbamate), 1401 (C–N, carbamate), 1368 (C(CH_3_)_3_), 1158 (C–O) cm^−1^.

*N*-Boc-Leucine (**6**). Reaction time: 18 h, yield: 91%, physical appearance: white powder, M.P: 82–86 °C, TLC system: (30% ethyl acetate/n-hexane, Stain, Ninhydrin), [α]_D_^25^: +8.13 (c = 10 mg/10 mL, CH_3_OH), FTIR (neat, v_max_): 2953 (C–H, CH(CH_3_)_2_, str), 1712 (C=O, acid), 1672 (C=O, carbamate), 1471 (C–N, carbamate), 1365 (C(CH_3_)_3_, 1169 (C–O) cm^−1^. Anal. calcd. for C_11_H_21_NO_4_ (231.29) C, 57.12; H, 7.52; N, 6.00. Found: C, 57.11; H, 7.49; N, 6.00.

#### 3.1.2. Synthesis of N-Boc-*O*-Benzyl-L-Serine (**7**)

*N*-Boc-L-Serine (**5**) (2 g, 10.0 mmol) was dissolved in 24 mL of DMF and stirred for 15 min. The NaH (0.26 g, 10.0 mmol) was then added to the reaction mixture at 0 °C and stirred till room temperature. Subsequently, benzyl bromide (1 eq, 10.0 mmol, 0.63 mL) was added dropwise, and the reaction mixture was stirred for 6 h at room temperature. After completion of the reaction, the solvent was removed under vacuum, and a residue was obtained that was dissolved in water and extracted three times with 10 mL of diethyl ether. The pH of the aqueous layer was adjusted to 1–2 using dilute KHSO_4_ solution and extracted with ethyl acetate, and the organic phase was dried over magnesium sulfate (MgSO_4_). The mixture was filtered, and the solvent was evaporated to obtain the pure product yield 62% [55].

Reaction time: 6 h, yield: 62%, physical appearance: brownish thick oil, TLC system: (20% CH_3_OH/CHCl_3_, Stain, Ninhydrin), [α]_D_^25^: +3.75 (c = 6 mg/10 mL CH_3_OH), FTIR (neat, v_max_): 3029 (Aromatic, C–H), 2928 (C–H, CH_2,_ str), 1712 (C=O, acid), 1454 (Aromatic, C=C), 1367 (C(CH_3_)_3_, 1236 (C–O, acid), 1158 (C–O, ether), 735 (para-substitution) cm^−1^.

#### 3.1.3. Synthesis of *O*-Benzyl-L-Tyrosine (**9**)

L-Tyrosine (**8**) (2 g, 11.0 mmol) was dissolved in 5.6 mL of 2N NaOH solution. Then, a solution of copper (II) sulfate pentahydrate (1 eq, 5.60 mmol, 1.4 g) in 5.6 mL of water was added in the reaction mixture and stirred for 30 min. It was then heated at 60 °C for 15 min, cooled to room temperature, and 40 mL of methanol was added. After it, more 2N NaOH was added. Afterward, benzyl bromide (11.78 mmol, 1.4 mL) was added, and the reaction was stirred at room temperature for a 4 h. The precipitates formed were separated by filtration and washed sequentially with a methanol/water mixture, pure methanol, 1N (HCl), distilled water, 1N (NH_4_OH), and finally with distilled water again. The product was obtained as a white solid yield of 60% [56].

Reaction time: 6 h, yield: 60%, physical appearance: white powder, M.P: 258–260 °C, TLC system: (30% ethyl acetate/n-hexane, Stain, Ninhydrin), [α]_D_^25^: −10.93 (c = 12 mg/10 mLCH_3_OH), FTIR (neat, v_max_): 3050 (Aromatic, C–H), 2978 (C–H, CH_3,_ str), 2953 (C–H, CH_2,_ str), 1737 (C=O, acid), 1455 (Aromatic, C=C), 1253 (C–O, acid), 1174 (C–O, ether), 870 (para-substitution) cm^−1^. Anal. calcd. for C_16_H_17_NO_3_ (271.32): C, 70.84; H, 6.42; N, 5.15. Found: C, 70.82; H, 6.41; N, 5.14.

#### 3.1.4. Synthesis of *N*-Boc-*O*-Benzyl-L-Tyrosine (**10**)

In a solution of dioxane and water (2:1, 44 mL), crude *O*-Benzyl-L-Tyrosine (**9**) (2 g, 7.37 mmol) was dissolved. To this solution, 1N NaOH (7.38 mL, 7.38 mmol) and NaHCO_3_ (0.59 g, 7.02 mmol) were added. The resulting mixture was stirred, and then (Boc)_2_O) (3.3 g, 15.53 mmol, 2 eq) was added at 0 °C. The reaction mixture was stirred for 15 h at room temperature. After the completion of reaction, the mixture was filtered to remove any undissolved solids. After concentrating the reaction mixture, ethyl acetate was added. The pH of the reaction mixture was then adjusted by 1–2 using aqueous potassium hydrogen sulfate and subsequently extracted with ethyl acetate. The organic layer was washed with brine, dried over magnesium sulfate, filtered, and concentrated using a rotary evaporator. The resulting product was obtained with a yield of 91% as a light-yellow gel [38].

Reaction time: 18 h, yield: 91%, physical appearance: brownish thick gel, TLC system: (30% ethyl acetate/n-hexane, Stain, Ninhydrin), [α]_D_^25^: +27.5 (c = 5 mg/10 mL CH_3_OH), FTIR (neat, ν_max_): 3052 (Aromatic, C–H), 2977 (C–H, CH_2,_ str), 1737 (C=O, acid), 1455 (Aromatic, C=C), 1251 (C–O, acid), 1176 (C–O, ether), 870 (para-substitution) cm^−1^. Anal. calcd. for C_21_H_25_NO_5_ (371.43): C, 67.88; H, 6.77; N, 3.78. Found: C, 67.85; H, 6.75; N, 3.77.

### 3.2. Synthesis of N-Boc-L-Leucine Diazoketone (***11***)

In a two-neck round-bottom flask dry THF (32.4 mL) and *N*-Boc-Leucine (**6**) (1.5 g, 6.48 mmol), dry Et_3_N (1.1 mL, 7.78 mmol, 1.2 eq), and ethyl chloroformate (0.9 mL, 9.7 mmol, 1.5 eq) were added under an inert atmosphere. The reaction mixture was stirred at −15 °C for 20 min. After it, diazomethane solution in ether was added until a bright-yellow color persisted for a longer period of time. The reaction mixture was then stirred at room temperature for 4 h. Upon completion of the reaction, a few drops of acetic acid were added to neutralize excess diazomethane. After rotary evaporation of the reaction mixture, ethyl acetate was added, and the organic layer was washed successively with solutions of sodium bicarbonate, ammonium chloride, and sodium chloride. The organic layer was then separated and evaporated to obtain the crude product. The crude product was further purified by silica gel column chromatography by using a 30% ethyl acetate/n-hexane solvent system, resulting in the isolation of the diazoketone with a good yield of 73% [38].

Reaction time: 6 h, yield: 73%, physical appearance: yellow solid, TLC system: (30% ethyl acetate/n-hexane, Stain, Ninhydrin), [α]_D_^25^: −13 (c = 2 mg/10 mL, CH_3_OH), FTIR (neat, ν_max_): 3315 (sec N–H, str), 2962 (C–H, CH_3_, str), 2934 (C–H, CH_2,_ str), 2102 (H-C=N_2_), 1703 (C=O, acid), 1672 (C=O, carbamate), 1626 (N–H, bending), 1365 (C(CH_3_)_3_, 1154 (C–O) cm^−1^. ^1^H NMR (300 MHz, DMSO): δ 6.1 (s, 1H), 4.0 (bs, 1H), 1.7–1.6 (m, 1H), 1.59–1.50 (m, 2H), 1.41 (s, 9H), 0.91 (d, *J* = 6.3 Hz, 6H).

### 3.3. Synthesis of N-Boc-L-Leucine-β-Methyl Ester (***12***)

Under an inert atmosphere at −25 °C, *N*-Boc-Leucine diazoketone (**11**) (0.5 g, 1.9 mmol) was dissolved in dry CH_3_OH (5.7 mL). Then, a solution of silver benzoate (66.9 mg, 0.29 mmol, 0.11 eq) in dry Et_3_N (0.77 mL, 5.6 mmol, 2.9 eq) was added. The mixture was then stirred in the dark at room temperature for 8 h. After completion of the reaction, the solvent evaporated, leaving behind a residue that was subsequently diluted with ethyl acetate. The resulting solution was washed with solutions of NaHCO_3_, NH_4_Cl, and sodium chloride NaCl. The organic layer was then dried over MgSO_4_, filtered, and the solvent was removed by rotary evaporation. The crude product obtained was further purified by silica gel column chromatography using a 30% ethyl acetate/n-hexane solvent system, resulting in the isolation of the pure β-methyl ester with a good yield of 71% [42].

Reaction time: 6 h, yield: 71%, physical appearance: colorless thick gel, TLC system: (30% ethyl acetate/n-hexane, Stain, Ninhydrin), [α]_D_^25^: −13.5 (c = 2 mg/10 mL, CH_3_OH), FTIR (neat, ν_max_): 3361 (sec N–H, str), 2955 (C–H, CH_3_, str), 2927 (C–H, CH_2,_ str), 1738 (C=O, ester), 1693 (C=O, carbamate), 1454 (C=C, Aromatic), 1365 (C(CH_3_)_3_, 1166 (C–O) cm^−1^. ^1^H NMR (300 MHz, DMSO): δ 4.2–4.1 (m, 1H), 3.62 (s, 3H), 2.40–2.36 (d, *J* = 6.9 Hz, 2H), 1.59–1.49 (m, 1H), 1.42 (s, 9H), 1.35–1.30 (m, 2H), 0.85 (d, *J* = 6.3 Hz, 6H).

### 3.4. Boc-Deprotection of N-Boc-L-Leucine-β-Methyl Ester (***13***)

Under an inert atmosphere at 0 °C, *N*-Boc-leucine-β-methyl ester (**12**) (300 mg, 1.15 mmol) was dissolved in dry CHCl_3_ (7 mL). Trifluoroacetic acid (TFA) (6.34 mmol, 0.52 mL) was then added to the reaction mixture, and it was stirred for 3 h. Upon completion of the reaction, the solvent was evaporated, and product was obtained in the form of its triflate salt [39].

Reaction time: 8 h, yield: 97%, physical appearance: dark-brown thick gel, TLC system: (50% ethyl acetate/n-hexane and 20% methanol/chloroform, Stain, Ninhydrin), [α]_D_^25^: −39.9 (c = 3 mg/10 mL, CH_3_OH), FTIR (neat, ν_max_): 3100 (sec N–H, str), 2940 (C–H, CH_3_, str), 2912 (C–H, CH_2,_ str), 1781 (C=O, ester), 1146 (C–O) cm^−1^.^1^H NMR (300 MHz, DMSO): δ 4.20–4.10 (m, 1H), 3.62 (s, 3H), 3.10–2.60 (d, *J* = 6.9 Hz, 2H), 1.59–1.49 (m, 1H), 1.40–1.30 (m, 2H), 1.50 (d, *J* = 7.5 Hz, 6H).

### 3.5. Synthesis of N-Boc-α-Glycine-β-Leucine Methyl Ester Dipeptide (***14***)

Under an inert atmosphere at 0 °C, Boc-glycine (**4**) (225 mg, 1.28 mmol, 1 eq) in dry CHCl_3_ (6 mL) was mixed with Boc-deprotected β-leucine-methyl ester trifluoroacetate (**13**) (350 mg, 1.28 mmol, 1 eq) in dry CHCl_3_ (5 mL) and Et_3_N (2.12 mL, 15.4 mmol, 6 eq). To this mixture, EDC HCl (295 mg, 1.53 mmol, 1.2 eq) and HOBt (208 mg, 1.54 mmol, 1.2 eq) were added. The reaction mixture was then allowed to stir at room temperature for 20 h. Upon completion of the reaction, the mixture was diluted with chloroform and washed with 1N HCl. The organic layer was further washed with solutions of sodium bicarbonate NaHCO_3_ and NaCl, dried over MgSO_4_, and concentrated using rotary evaporation. The crude product was obtained and purified by silica gel column chromatography using a solvent system of 10% methanol/chloroform, resulting in a good yield 72% [57].

Reaction time: 18 h, yield: 72%, physical appearance: brown sticky thick gel, TLC system: (50% ethyl acetate/n-hexane and 10% CH_3_OH/CHCl_3_, Stain, Ninhydrin), [α]_D_^25^: +1.19 (c = 3 mg/10 mL, CH_3_OH), FTIR (neat, ν_max_): 3306 (sec N–H, str), 2955 (C–H, CH_3_, str), 2928 (C–H, CH_2,_ str), 1718 (C=O, ester), 1655 (C=O, amide), 1520 (N–H bending, amide), 1365 (C(CH_3_)_3_, 1248 (C–O, ester), 1168 (C–O, carbamate) cm^−1^. ^1^H NMR (300 MHz, CDCl_3_): δ 6.85 (m, 1H), 4.2 (m, 1H), 3.81 (s, 3H), 2.57 (d, *J* = 5.4 Hz, 2H), 2.1–2.0 (m, 1H), 1.42 (s, 9H), 0.85 (d, *J* = 6.0 Hz, 6H), ^13^C NMR (75 MHz, CDCl_3_) δppm: 172.86, 170.59, 155.84, 79.20, 46.41, 43.75, 41.47, 39.20, 28.39, 28.30, 28.21, 25.29, 22.44, 13.80.

### 3.6. Boc-Deprotection of N-Boc-α-Glycine-β-Leucine Methyl Ester Dipeptide (***15***)

Under an inert atmosphere at 0 °C, Boc-α-glycine-β-leucine methyl ester (**14**) (160 mg, 0.507 mmol) was dissolved in dry dichloromethane (7 mL). Trifluoroacetic acid (0.31 mL, 4.1 mmol, 8.0 eq) was then added to the solution, and the reaction mixture was stirred for 3 h. The solvent was rotary-evaporated after reaction completion, resulting in Boc-deprotected dipeptide [39].

Reaction time: 6 h, physical appearance: thick brown oil, FTIR (neat, ν_max_): 2951 (C–H, CH_3_, str), 1710 (C=O, ester), 1670 (NH-str), 1259 (C–O, ester).

### 3.7. Synthesis of N-Boc-O-Benzyl-α-Serine-β-Leucine-Methyl Ester Dipeptide (***16***)

Under a nitrogen atmosphere at 0 °C, *N*-Boc-*O*-benzyl-α-serine (**7**) (542 mg, 1.83 mmol, 1 eq) in dry CHCl_3_ (7.5 mL) was added to Boc-deprotected β-leucine-methyl ester triflate salt (**13**) (500 mg. 1.83 mmol, 1 eq) in dry CHCl_3_ (7 mL), along with Et_3_N (2.04 mL, 14.6 mmol, 8 eq). Subsequently, EDC HCl (422 mg, 2.21 mmol, 1.2 eq) and HOBt (298 mg, 2.21 mmol, 1.2 eq) were added. The reaction mixture was then stirred at room temperature for 20 h. After completion of the reaction, the mixture was diluted with chloroform and washed with 1N HCl. The organic layer was further washed with solutions of NaHCO_3_ and NaCl, dried over MgSO_4_, and concentrated using rotary evaporation to obtain the crude product. This crude product was then purified by silica gel column chromatography using a solvent system of 40% ethyl acetate/n-hexane, resulting in the isolation of the pure product with a yield of 68% [57].

Reaction time: 18 h, yield: 68%, physical appearance: yellow thick gel, TLC system: (40% ethyl acetate/n-hexane and 10% CH_3_OH/CHCl_3_, Stain, Ninhydrin), [α]_D_^25^: +1.50 (c = 1 mg/10 mL, CH_3_OH), FTIR (neat, ν_max_): 3356 (sec N–H, str), 3021 (C–H, Aromatic), 2925 (C–H, CH_3_, str), 2878 (C–H, CH_2,_ str), 1731 (C=O, ester), 1615 (C=O, amide), 1520 (N–H bending, amide), 1372 (C(CH_3_)_3_, 1262 (C–O, ester), 1130 (C–O, carbamate) cm^−1^. ^1^H NMR (300 MHz, CDCl_3_): 7.25–7.30 (m, 5H), 6.5 (d, *J* = 8.0 Hz, 1H), 4.51 (m, 2H), 4.45 (d, (NH), *J* = 6.5Hz, 1H,), 4.10 (m, 1H), 3.75 (m, 2H), 3.56 (s, 3H), 2.51 (dd, *J* = 10.0 Hz, 6.5 Hz, 1H), 2.45 (dd, *J* = 11.0 Hz, 5.5 Hz, 1H), 1.40 (s, 9H), 0.90–0.85 (d, *J* = 8 Hz, 6H), ^13^C NMR (75 MHz, CDCl_3_) δppm: 172.83, 171.02, 156.12, 137.73, 128.32, 128.18, 128.10, 72.36, 69.63, 69.58, 53.84, 46.10, 41.43, 39.32, 25.28, 22.43, 22.11, 13.08.

### 3.8. Synthesis of N-Boc-O-Benzyl-α-Tyrosine-α-Glycine-β-Leucine Methyl Ester Tripeptide (***17***)

To the mixture of *N*-Boc-*O*-benzyl-tyrosine (**10**) (408 mg, 1.10 mmol, 1 eq) in dry chloroform (8 mL) under nitrogen at 0 °C, Boc-deprotected-α-glycine-β-leucine methyl ester trifluoroacetate (**15**) (329 mg, 1.10 mmol, 1 eq) in dry chloroform (7 mL) and Et_3_N (1.22 mL, 8.7 mmol, 8 eq), EDC (252 mg, 1.31 mmol, 1.2 eq) and HOBt (178 mg, 1.31 mmol, 1.2 eq) were added. The mixture was allowed to stir at room temperature for 20 h. After the reaction, the mixture was diluted with chloroform and washed with 1N HCl. The organic layer was washed with NaHCO_3_ and NaCl solutions dried over MgSO_4_ and rotary-evaporated for a crude product, purified by silica gel column chromatography (10% CH_3_OH/CHCl_3_); the product yield was 65% [57].

Reaction time: 18 h, yield: 65%, physical appearance: brownish thick gel, TLC system: (50% ethyl acetate/n-hexane and 10% CH_3_OH/CHCl_3_, Stain, Ninhydrin), [α]_D_^25^: +1.21 (c = 3 mg/10 mL, CH_3_OH), FTIR (neat, ν_max_): 3337 (sec N–H, str), 3005 (C–H, aromatic), 2925 (C–H, CH_3_, str), 2889 (C–H, CH_2,_ str), 1729 (C=O, ester), 1658 (C=O, amide), 1504 (N–H bending, amide), 1367 (C(CH_3_)_3_, 1261 (C–O, ester), 1120 (C–O, carbamate), 742 (mono-substitution)cm^−1^. ^1^H NMR (300 MHz, CDCl_3_): δ 8.2 (d, *J* = 8.4 Hz, 1H), 8.0 (d, *J* = 8.4 Hz, 1H), 7.8–7.7 (m, 2H), 7.6–7.5 (m, 2H), 7.48–7.35 (m, 4H), 7.1 (d, *J* = 8.7 Hz, 1H), 6.9 (d, *J* = 8.7 Hz, 1H), 5.2 (d, *J* = 5.4 Hz, 2H), 4.30–4.25 (m, 1H), 4.2–4.1 (m, 1H), 3.7 (s, 2H), 3.65 (s, 3H), 2.6 (d, *J* = 5.7 Hz, 2H), 1.60–1.49 (m, 1H), 1.5 (s, 9H), 1.34–1.30 (m, 2H), 0.92 (d, *J* = 6.3 Hz, 6H). ^13^C NMR (75 MHz, CDCl_3_) δppm: 171.80, 170.27, 157.45, 155.61, 151.43, 136.74, 132.03, 130.61, 128.48, 128.11, 127.90, 115.20, 79.45, 69.84, 55.60, 54.72, 48.42, 43.02, 41.30, 37.88, 37.72, 28.30, 25.28, 22.45.

### 3.9. α-Amylase Inhibition Assay

The reported procedure [58] was applied to investigate the α-amylase potential of test samples. Briefly, 15 µL phosphate buffer (pH 6.8) was drained into 96-well plate; later, 25 µL of alpha amylase enzyme (0.14 U/mL), 10 µL of fractions and refractions (4 mg/mL in DMSO), and 40 µL starch solution (2 mg/mL in potassium phosphate buffer) were added periodically. Incubation of samples was performed for 30 min at 50 °C. Thereupon, 20 µL of 1M HCl and lastly 90 µL iodine reagent (5 mM iodine, 5 mM potassium iodide) were combined in the respective wells. The negative control represented 100% enzyme activity and contained no test sample. Acarbose was employed as a positive control with a concentration range of 5–200 µg/mL. Blanks represented wells without test samples and enzymes. The results were taken at 540 nm and were measured using a microplate reader. The tested samples used for the compounds were 1 mg/mL of compound solution.

% Enzyme Inhibition = OD (s) − OD (n) ÷ OD (b) × 100

where

OD (s) = absorbance value of test sample;

OD (n) = absorbance of negative control;

OD (b) = absorbance of the blank.

## Figures and Tables

**Table 1:** α-Amylase inhibition results for synthesized short α-/β-mixed peptides.

Samples	% Inhibition
*N*-(Boc)-*O*-(Bz)-α-Ser-β-Leu–OCH_3_ (**16**)	45.22
N(Boc)-*O*-(Bz)-α-Tyr-α-Gly-β-Leu–OCH_3_ (**17**)	17.05
*N*-(Boc)-Gly-β-Leu–OCH_3_ (**14**)	18.51

Positive control = 15.78%.

## Data Availability

Data is contained within the article or Supplementary Materials.

## Supplementary Materials

The following supporting information can be downloaded at https://www.mdpi.com/article/10.3390/molecules29174028/s1. Figure S1: FTIR Spectrum of *N*-Boc-L-Glycine; Figure S2: FTIR Spectrum of *N*-Boc-L-Serine; Figure S3: FTIR Spectrum of *N*-Boc-L-Leucine; Figure S4: Spectrum of *N*-Boc-*O*-benzyl-L-Serine; Figure S5: FTIR Spectrum of O-Benzyl-L-Tyrosine; Figure S6: FTIR Spectrum of *N*-Boc-*O*-Benzyl-L-Tyrosine; Figure S7: FTIR Spectrum of *N*-Boc-L-Leucine Diazoketone; Figure S8: ^1^H NMR Spectrum of *N*-Boc-L-Leucine Diazoketone; Figure S9: ^1^H NMR Spectrum (Extended) of *N*-Boc-L-Leucine Diazoketone; Figure S10: FTIR Spectrum of *N*-Boc-L-Leucine β-Methyl Ester; Figure S11: ^1^H NMR Spectrum of *N*-Boc-L-Leucine β-Methyl Ester; Figure S12: ^1^H NMR Spectrum (Extended) of *N*-Boc-L-Leucine β-Methyl Ester; Figure S13: FTIR Spectrum of L-Leucine-β-Methyl Ester Trifluoroacetate (Salt); Figure S14: ^1^H NMR Spectrum of L-Leucine-β-Methyl Ester Trifluoroacetate (Salt); Figure S15: FTIR Spectrum of *N*-Boc-Glycine-β-Leucine Methyl Ester Dipeptide; Figure S16: ^1^H NMR Spectrum of α-Boc-Glycine-β-Leucine Methyl Ester Dipeptide; Figure S17: ^1^H NMR Spectrum (Extended) of α-Glycine-β-Leucine Methyl Ester Dipeptide; Figure S18: 13C NMR Spectrum of α-Glycine-β-Leucine Methyl Ester Dipeptide; Figure S19: FTIR Spectrum of *N*-Boc-*O*-Bz-Serine-β-Leucine Dipeptide; Figure S20: ^1^H NMR Spectrum of *N*-Boc-*O*-Bz-Serine-β-Leucine Dipeptide; Figure S21: 13C NMR Spectrum of *N*-Boc-*O*-Bz-Serine-β-Leucine Dipeptide; Figure S22: FTIR Spectrum of TFA-Gly-β-Leucine OCH3. Figure S23: FTIR Spectrum of *N*-Boc-*O*-Bz-L-Tyrosine-L-Glycine-β-Leucine methyl Ester Tripeptide (17); Figure S24: ^1^H NMR Spectrum of α-Tyrosine-α-Glycine-β-Leucine Methyl Ester Tripeptide (17); Figure S25: ^1^H NMR Spectrum (Extended) of α-Tyrosine-α-Glycine-β-Leucine methyl Ester (17); Figure S26: 13C NMR Spectrum of α-Tyrosine-α-Glycine-β-Leucine methyl Ester (17).
