# Supplementary material for: A Convenient Synthesis of Short α-/β-Mixed Peptides as Potential α-Amylase Inhibitors
Source: Molecules. 2024 Aug 26;29(17):4028. doi: 10.3390/molecules29174028 (PMC11396456; doi:10.3390/molecules29174028)
Supplement: Supplementary file 1 [file molecules-29-04028-s001.zip › molecules-3133936-supplementary.pdf]

Supporting Information for the article titled as "**A Convenient Synthesis of Short α,β-Mixed Peptides as Potential α-Amylase Inhibitors**".

Figure S-1, **FTIR Spectrum of N-Boc-L-Glycin(4)** 2
Figure S-2, FTIR Spectrum of N-Boc-L-Serine (5) 2
Figure S-3, FTIR Spectrum of N-Boc-L-Leucine (6) 3
Figure S-4, Spectrum of N-Boc-O-benzyl-L-Serine (7) 3
Figure S-5, FTIR spectrum of *O*-Benzyl-L-Tyrosine (9) 4
Figure S-6, FTIR Spectrum of N-Boc-O-Benzyl-L-Tyrosine (10) 4
Figure S-7, **FTIR Spectrum of N-Boc-L-Leucine Diazoketone (11)** 5
Figure S-8, ^{1}H NMR Spectrum of N-Boc-L-Leucine Diazoketone (11) 5
Figure S-9, ^{1}H NMR Spectrum (Extended) of N-Boc-L-Leucine Diazoketone (11) 6
Figure S-10, FTIR Spectrum of N-Boc-L-Leucine β-Methyl Ester (12) 6
Figure S-11, ^{1}H NMR Spectrum of N-Boc-L-Leucine β-Methyl Ester (12) 7
Figure S-12 ^{1}H NMR Spectrum (Extended) of N-Boc-L-Leucine β-Methyl Ester (12) 7
Figure S-13, FTIR Spectrum of L-Leucine-β-Methyl Ester Trifluoroacetate (Salt) (13) 8
Figure S-14 ^{1}H NMR Spectrum of L-Leucine-β-Methyl Ester Trifluoroacetate (Salt) (13) 8
Figure S-15, FTIR Spectrum of N-Boc-Glycine-β-Leucine Methyl Ester Dipeptide (14) 9
Figure S-16, ^{1}H NMR Spectrum of α-Boc-Glycine-β-Leucine Methyl Ester Dipeptide (14) 9
Figure S17, ^{1}H NMR Spectrum (Extended) of α-Glycine-β-Leucine Methyl Ester Dipeptide (14) ... 10
Figure S-18, ^{13}C NMR Spectrum of α-Glycine-β-Leucine Methyl Ester Dipeptide (14) 11
Figure S-19, FTIR Spectrum of N-Boc-O-Bz-Serine-β-Leucine Dipeptide (16) 11
Figure S-20, ^{1}HNMR Spectrum of N-Boc-O-Bz-Serine-β-Leucine Dipeptide (16) 12
Figure S-21, ^{13}C NMR Spectrum of N-Boc-O-Bz-Serine-β-Leucine Dipeptide (16) 13
Figure S- 22, FTIR Spectrum of TFA-Gly-β-Leucine OCH_3 (16A) 14

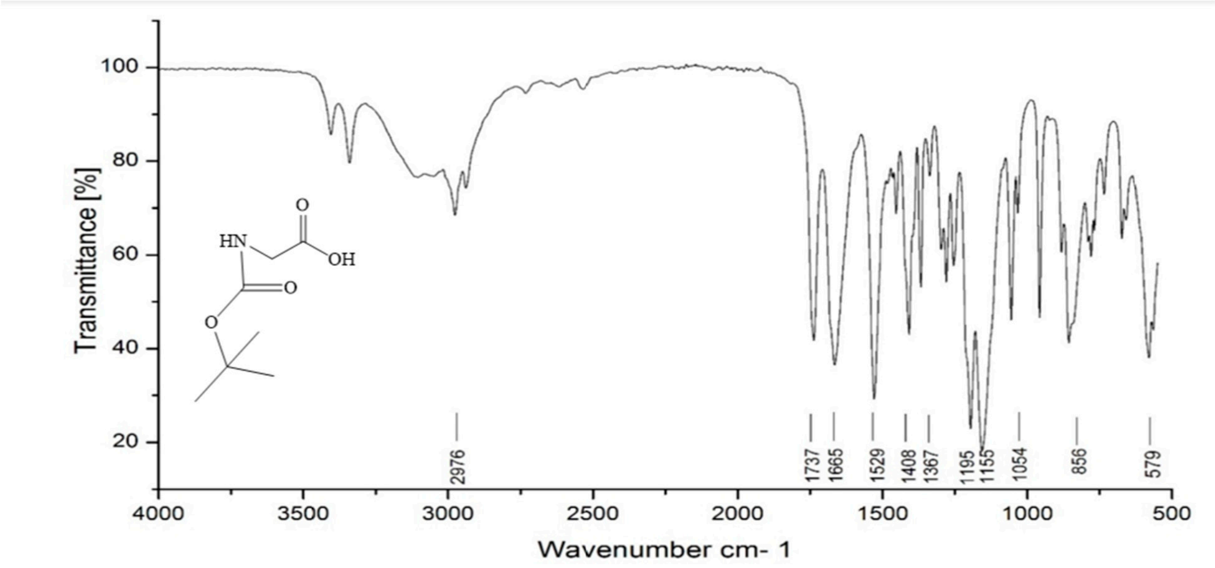

Figure S1. **FTIR Spectrum of N-Boc-L-Glycin(4)**

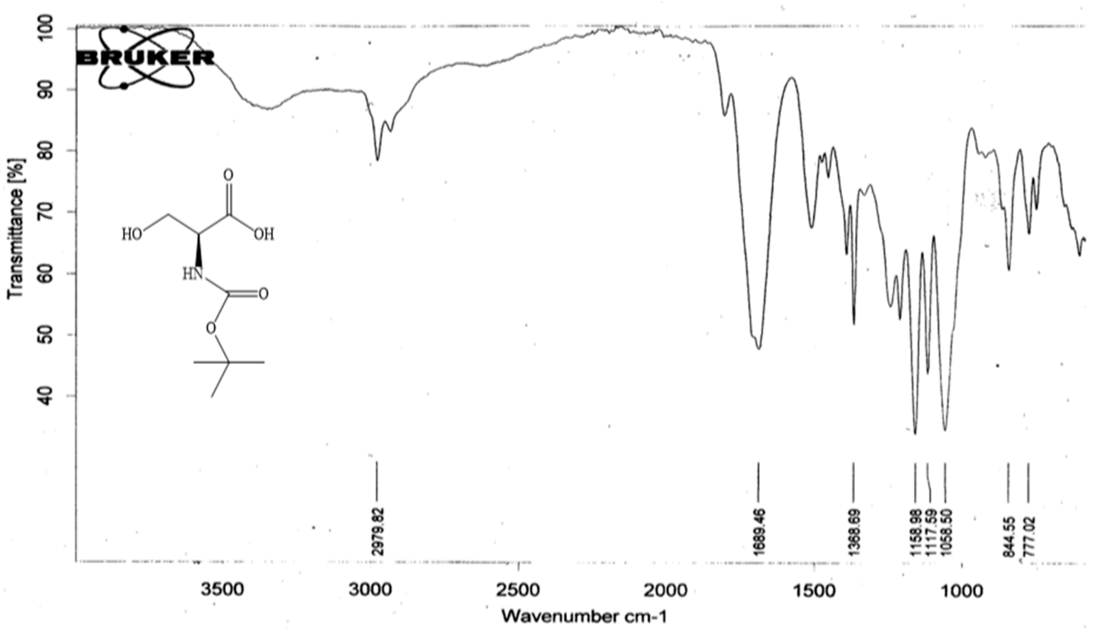

Figure S2. FTIR Spectrum of N-Boc-L-Serine (5)

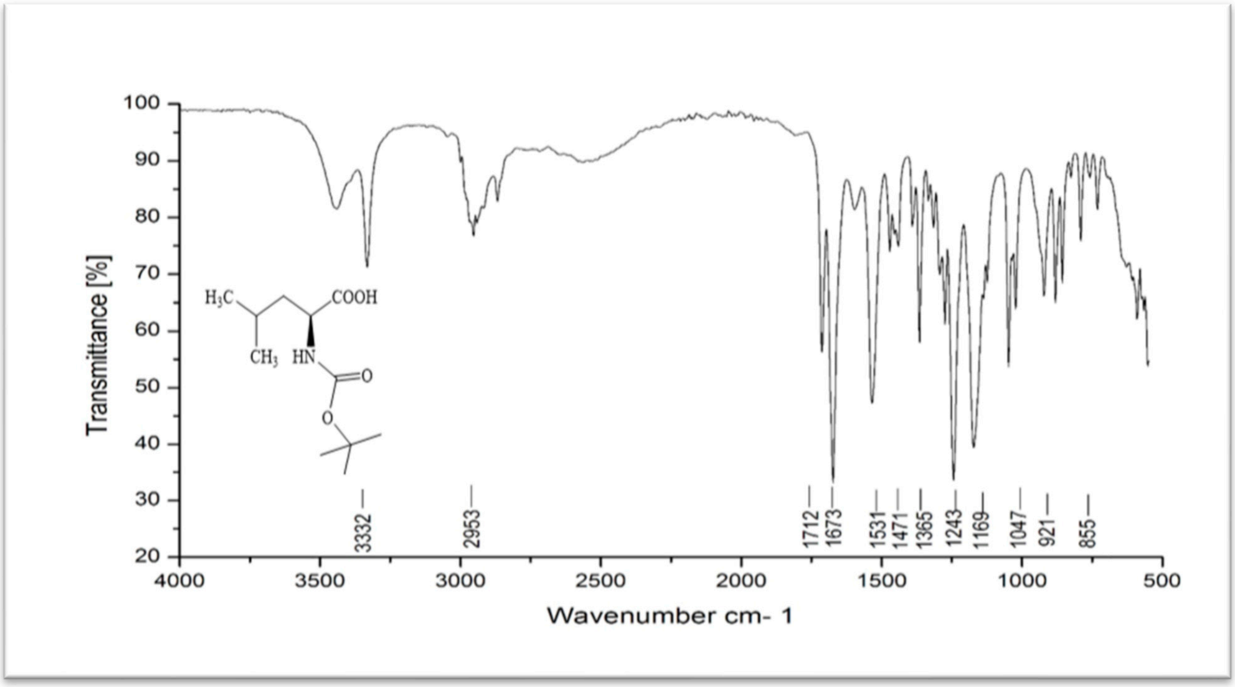

Figure S3. FTIR Spectrum of N-Boc-L-Leucine (6)

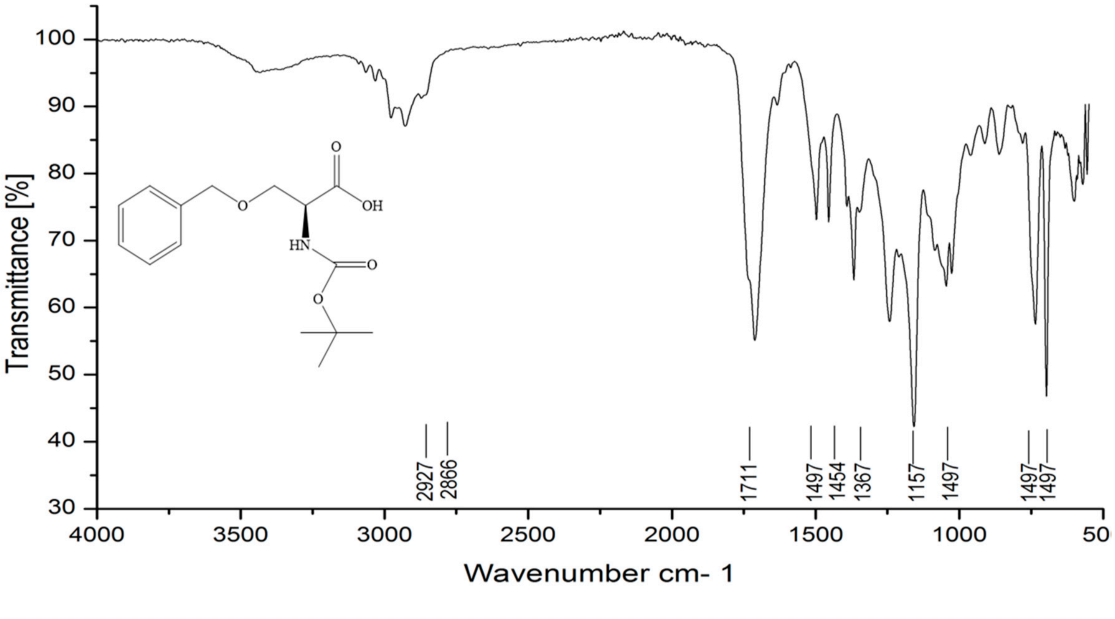

Figure S4. Spectrum of N-Boc-O-benzyl-L-Serine (7)

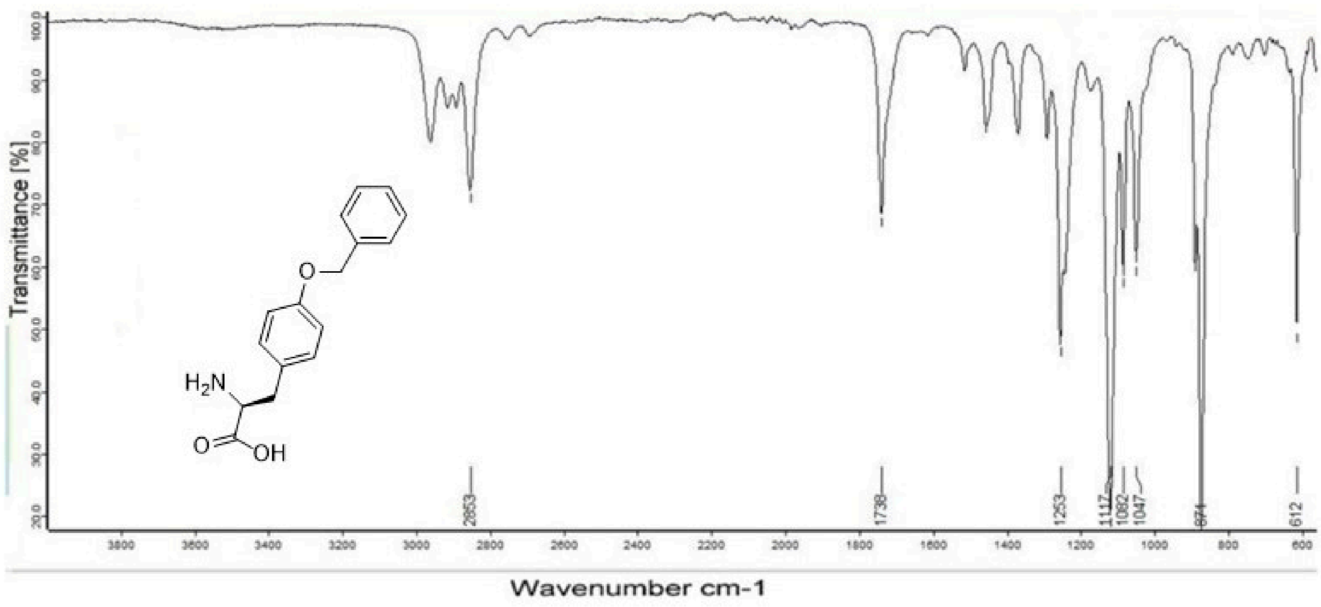

Figure S5. FTIR spectrum of *O*-Benzyl-L-Tyrosine (9)

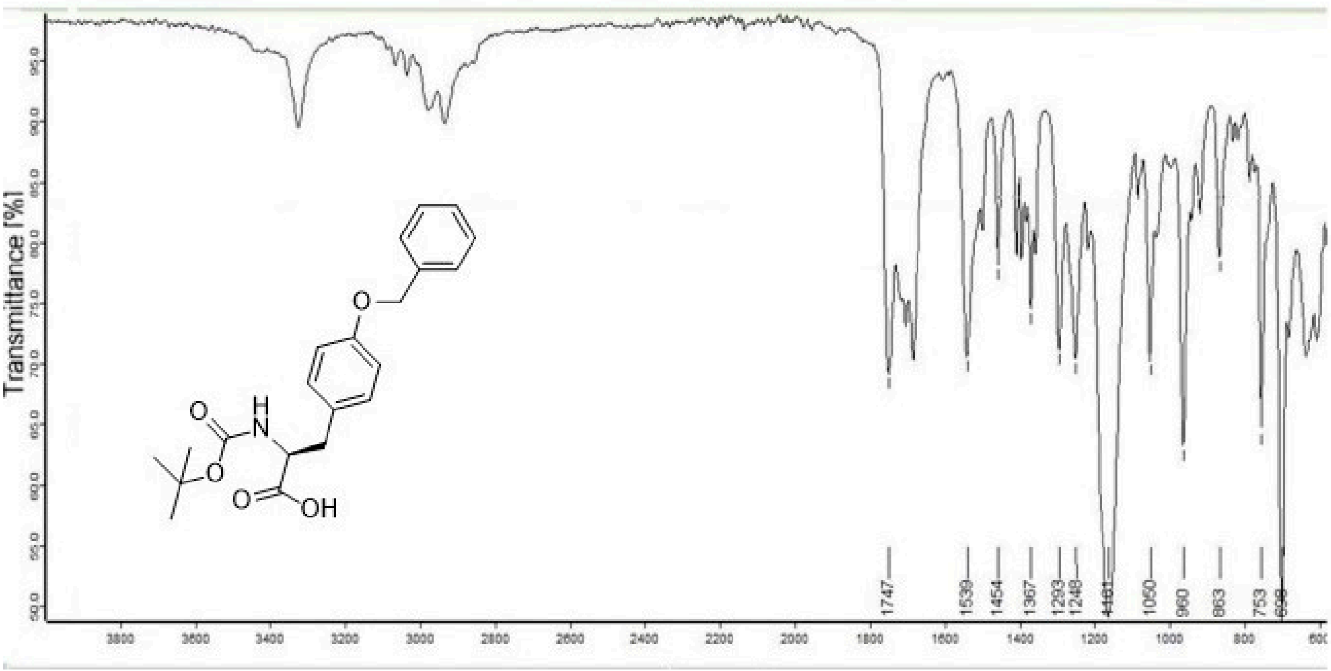

Figure S6. FTIR Spectrum of *N*-Boc-*O*-Benzyl-L-Tyrosine (10)

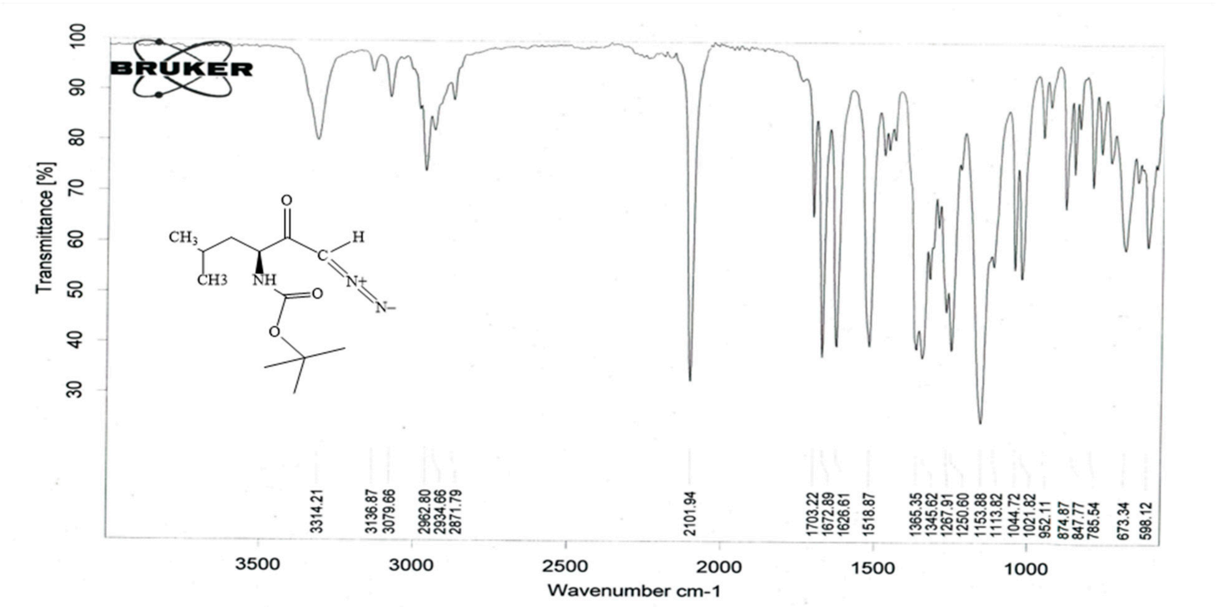

Figure S7. **FTIR Spectrum of *N*-Boc-L-Leucine Diazoketone (11)**

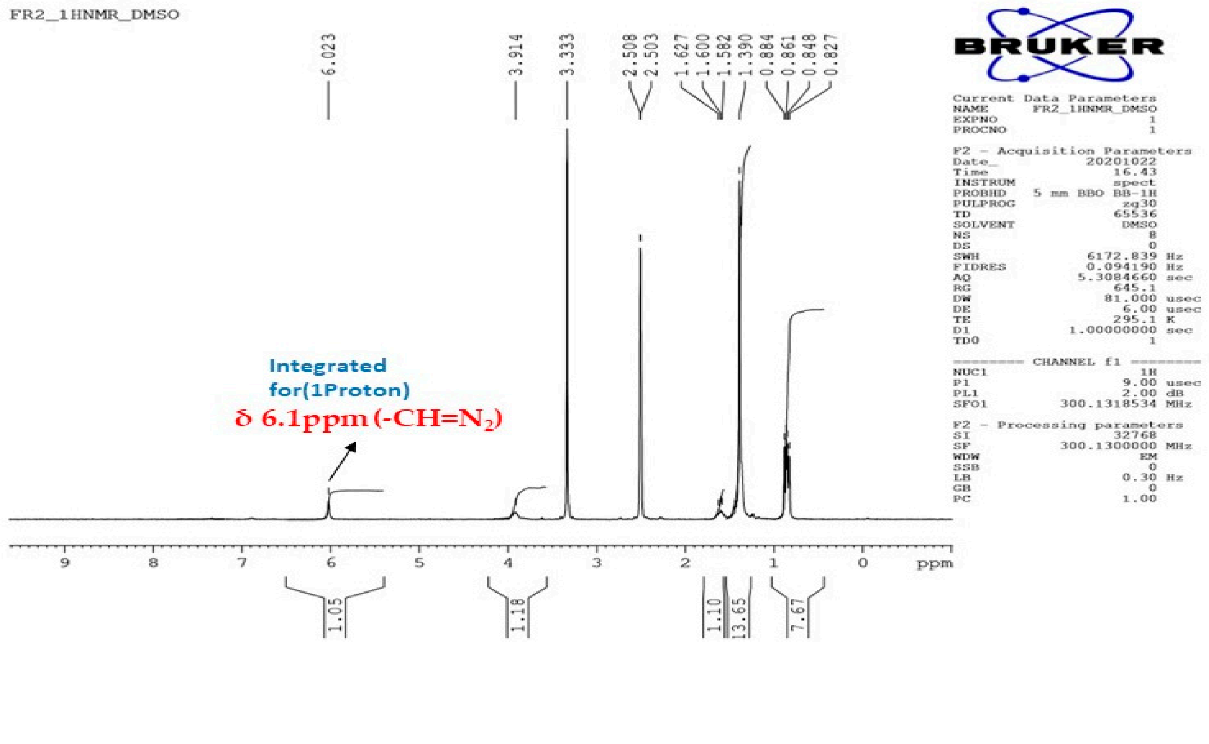

Figure S8. ^{1}H NMR Spectrum of *N*-Boc-L-Leucine Diazoketone (11)

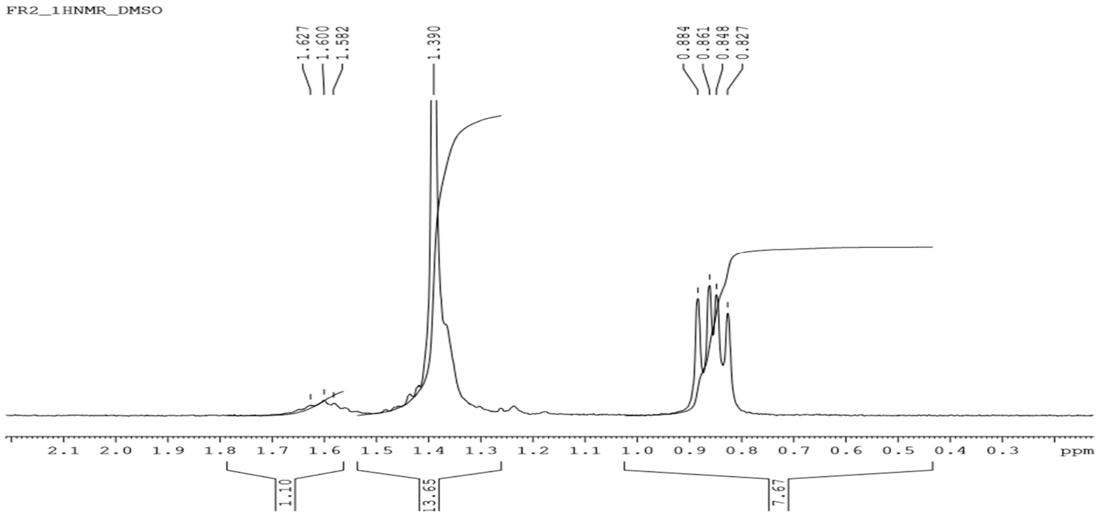

Figure S9. ^{1}H NMR Spectrum (Extended) of N-Boc-L-Leucine Diazoketone (11)

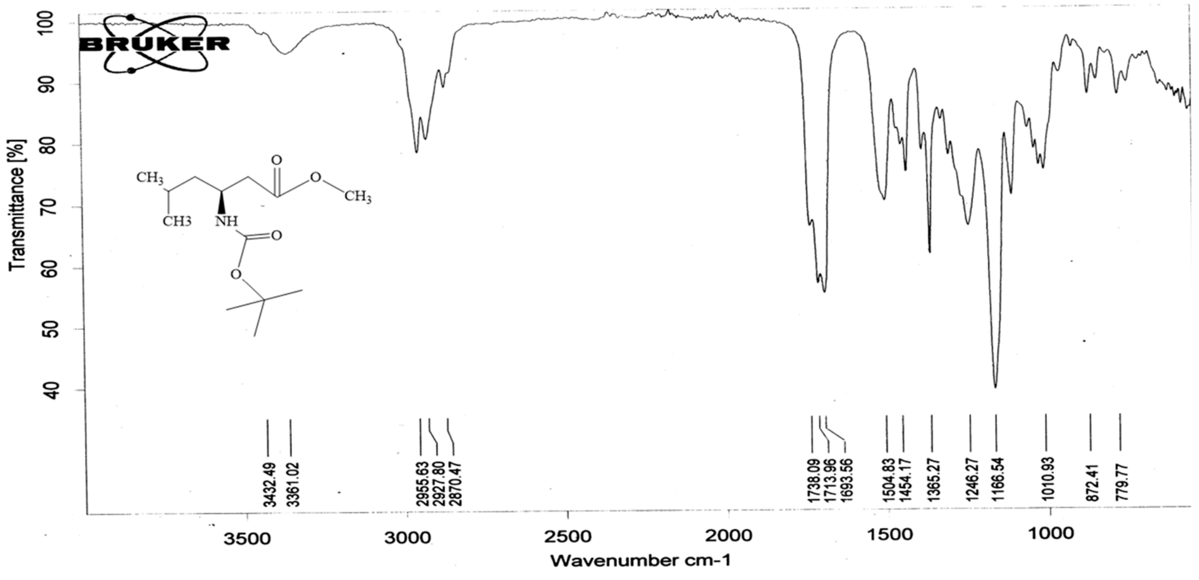

Figure S10. FTIR Spectrum of *N*-Boc-L-Leucine β-Methyl Ester (12)

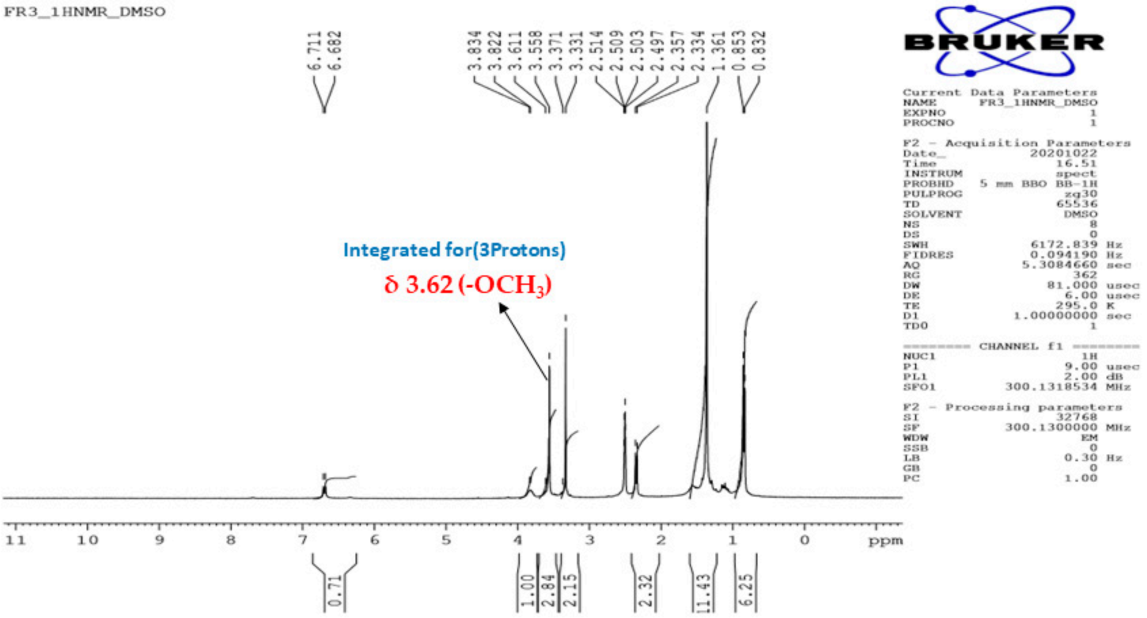

Figure S11. ^{1}H NMR Spectrum of N-Boc-L-Leucine β-Methyl Ester (12)

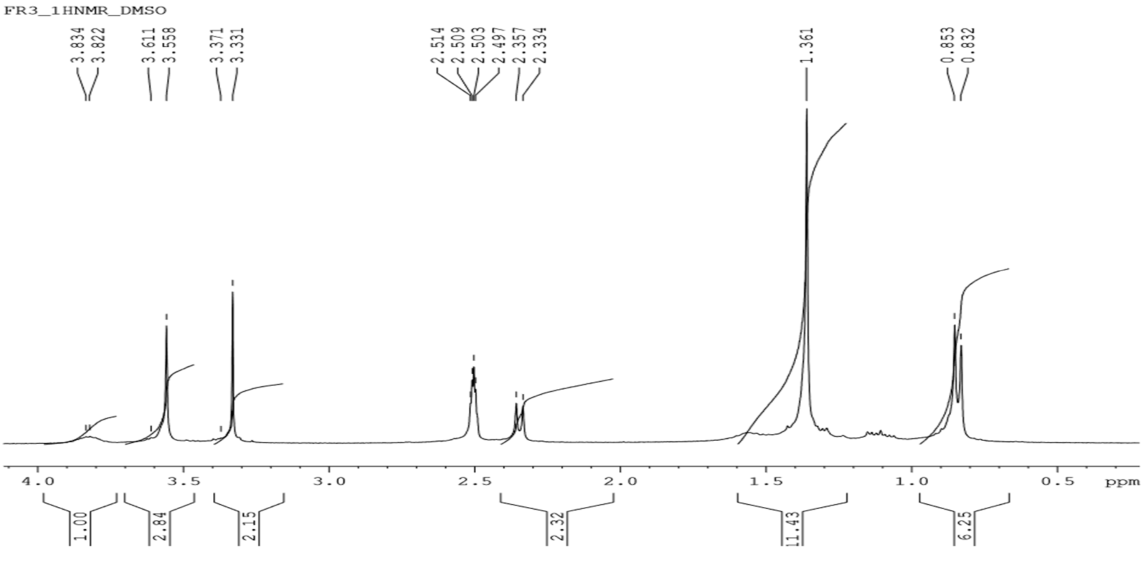

Figure S12. ^{1}H NMR Spectrum (Extended) of N-Boc-L-Leucine β-Methyl Ester (12)

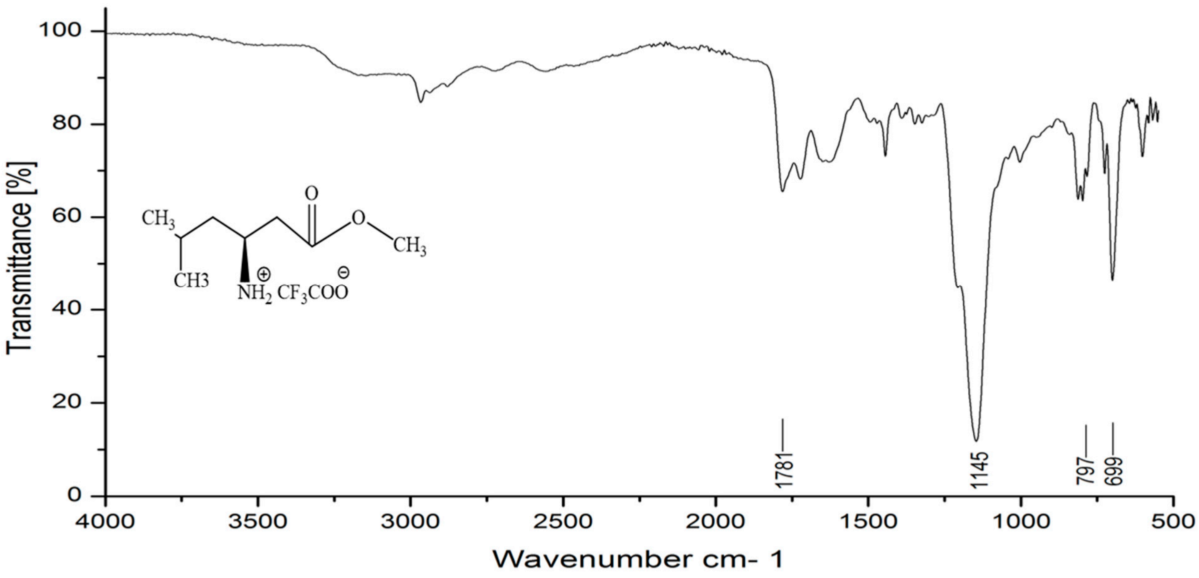

Figure S13. FTIR Spectrum of L-Leucine-β-Methyl Ester Trifluoroacetate (Salt) (13)

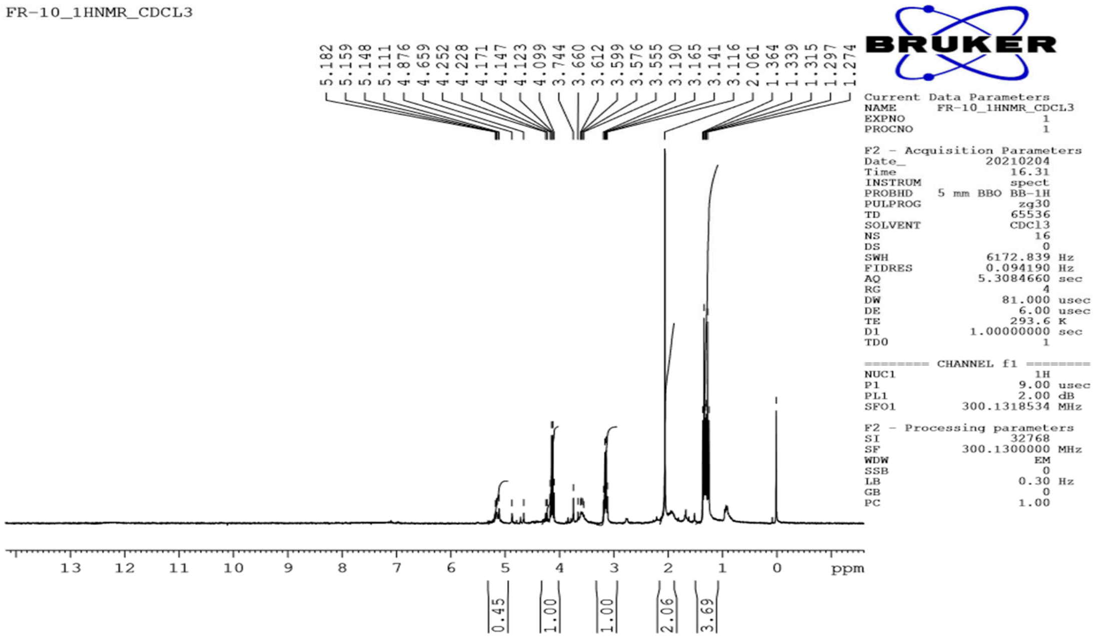

Figure S14. ^{1}H NMR Spectrum of L-Leucine-β-Methyl Ester Trifluoroacetate (Salt) (13)

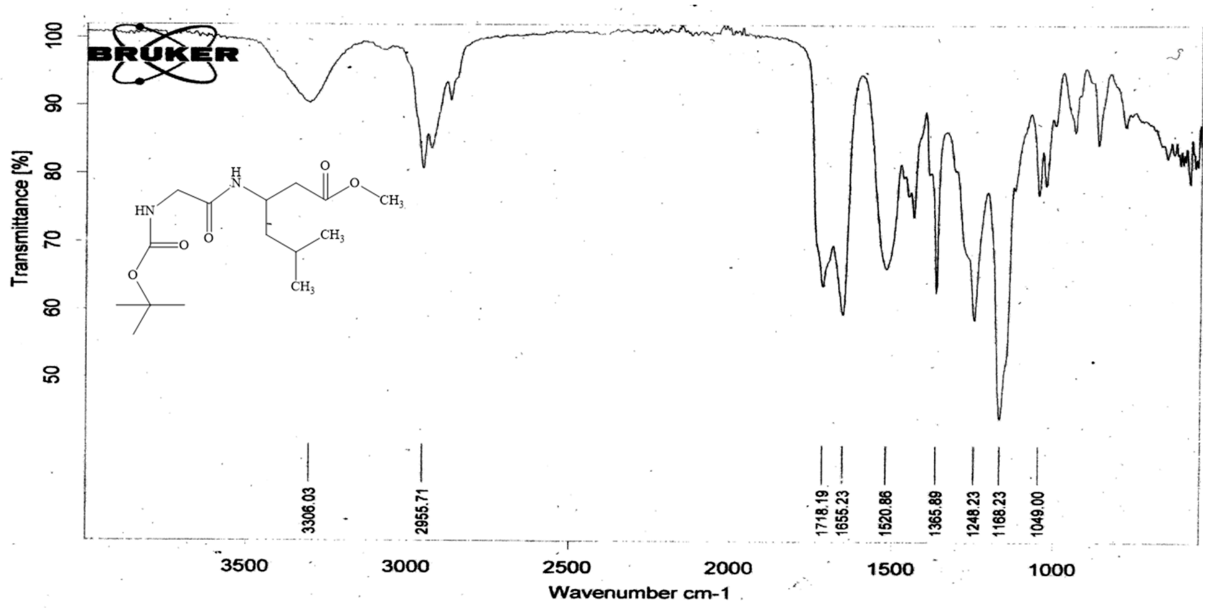

Figure S15. FTIR Spectrum of N-Boc-Glycine-β-Leucine Methyl Ester Dipeptide (14)

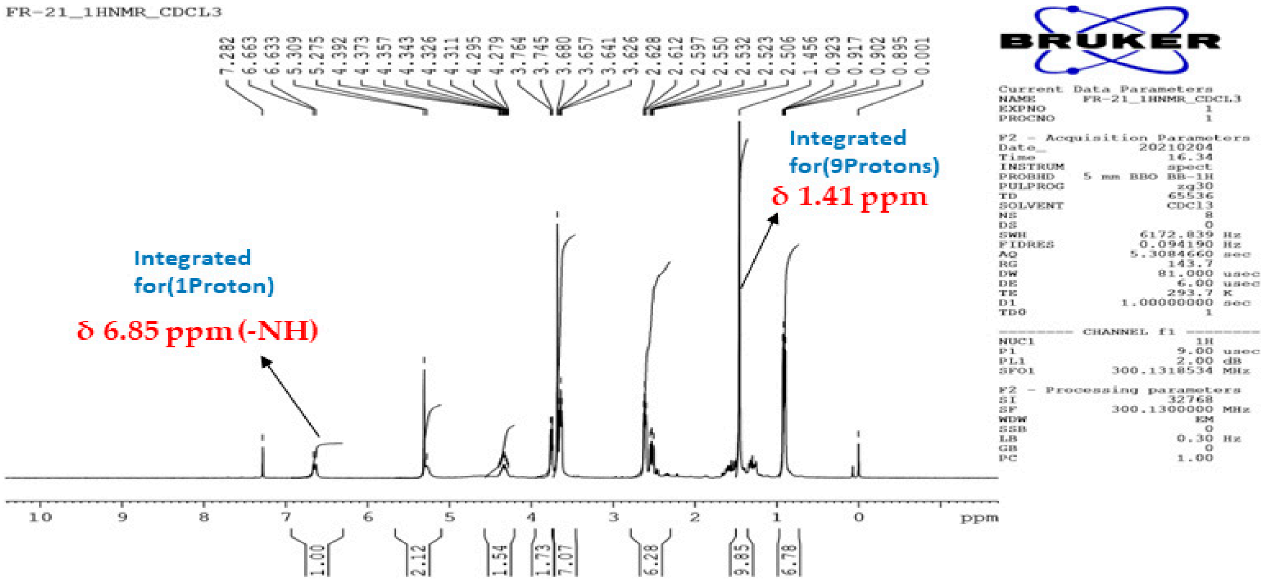

Figure S16. ^{1}H NMR Spectrum of α-Boc-Glycine-β-Leucine Methyl Ester Dipeptide (14)

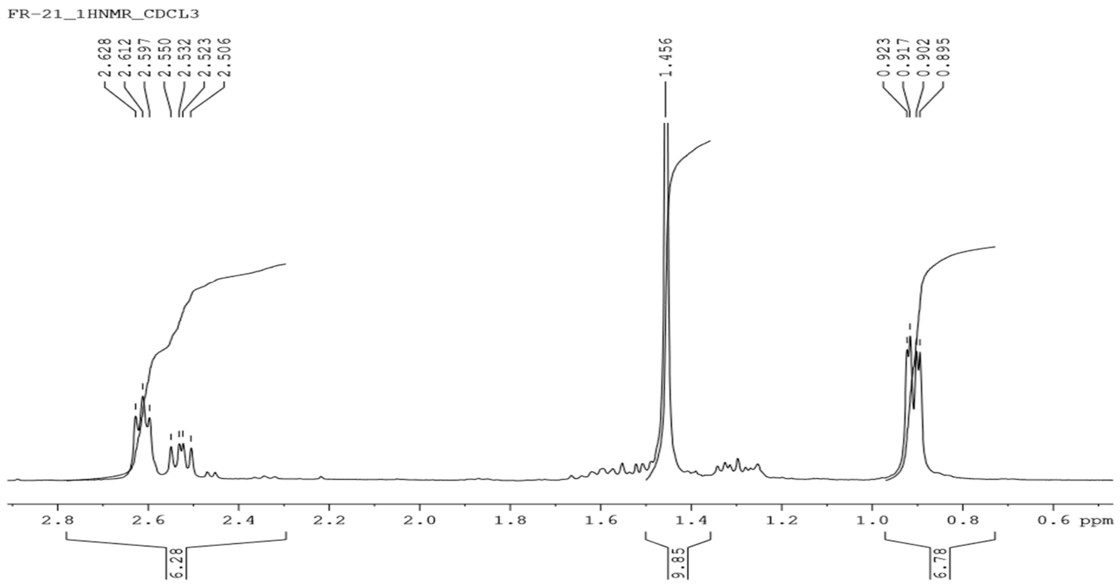

Figure S17. ¹H NMR Spectrum (Extended) of α-Glycine-β-Leucine Methyl Ester Dipeptide (14)

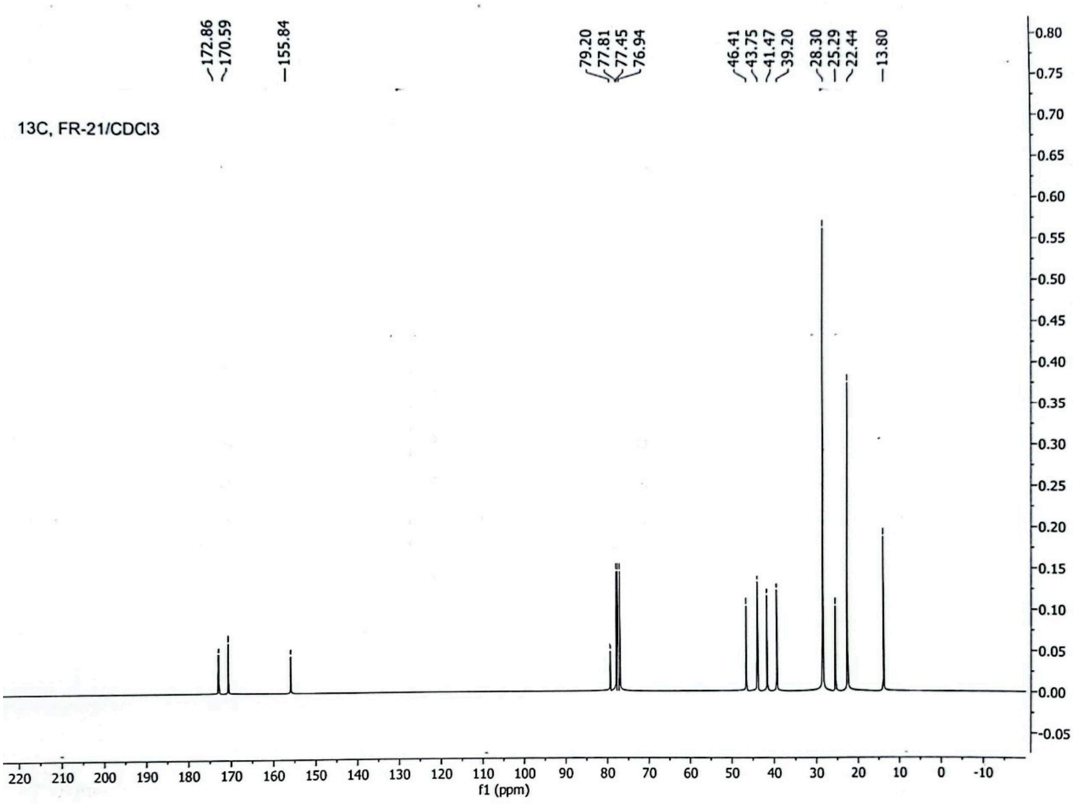

Figure S18. ^{13}C NMR Spectrum of α-Glycine-β-Leucine Methyl Ester Dipeptide (14)

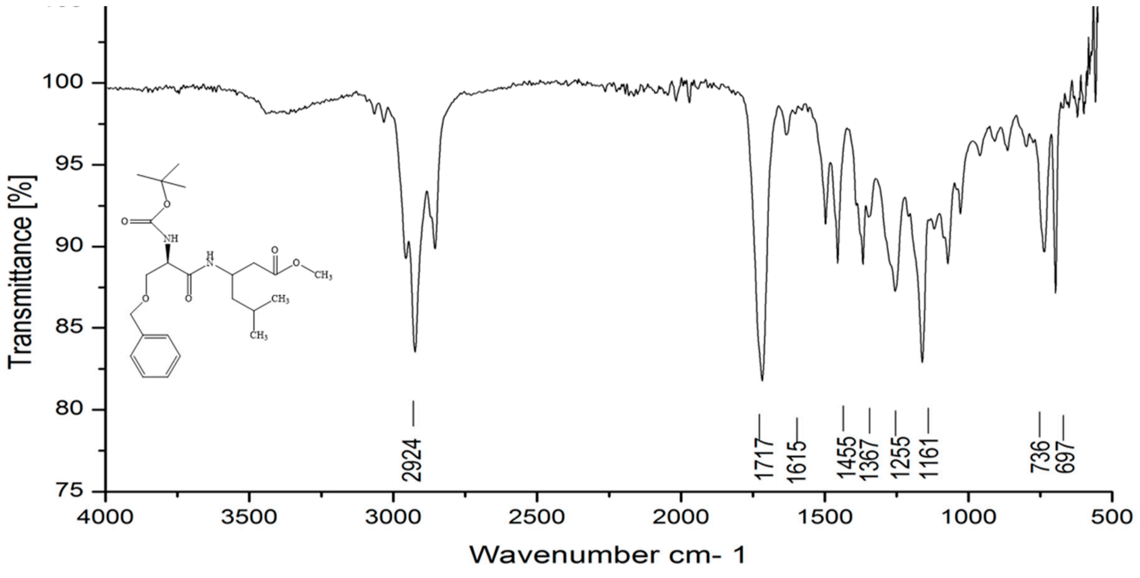

Figure S19. FTIR Spectrum of N-Boc-O-Bz-Serine-β-Leucine Dipeptide (16)

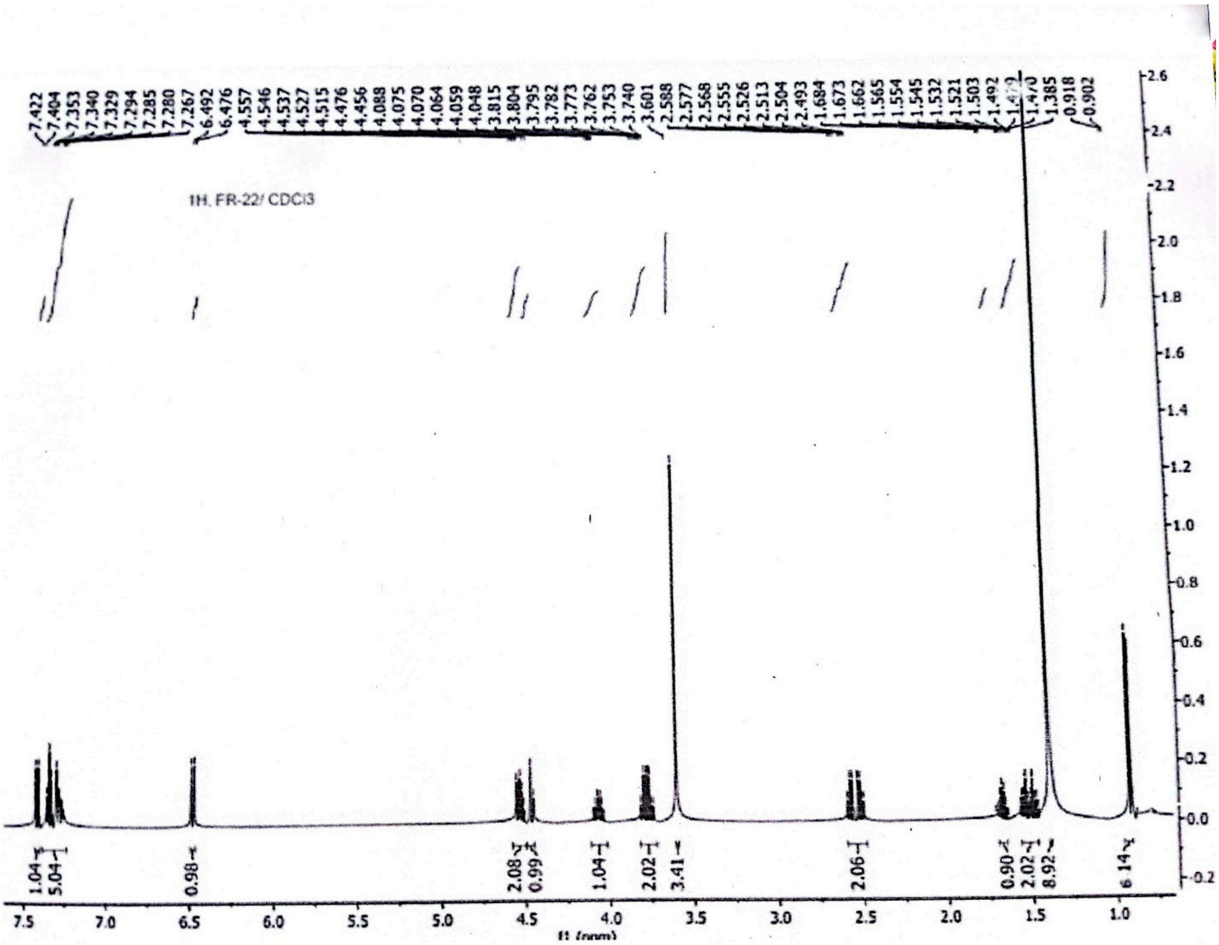

Figure S20. ^{1}HNMR Spectrum of N-Boc-O-Bz-Serine-β-Leucine Dipeptide (16)

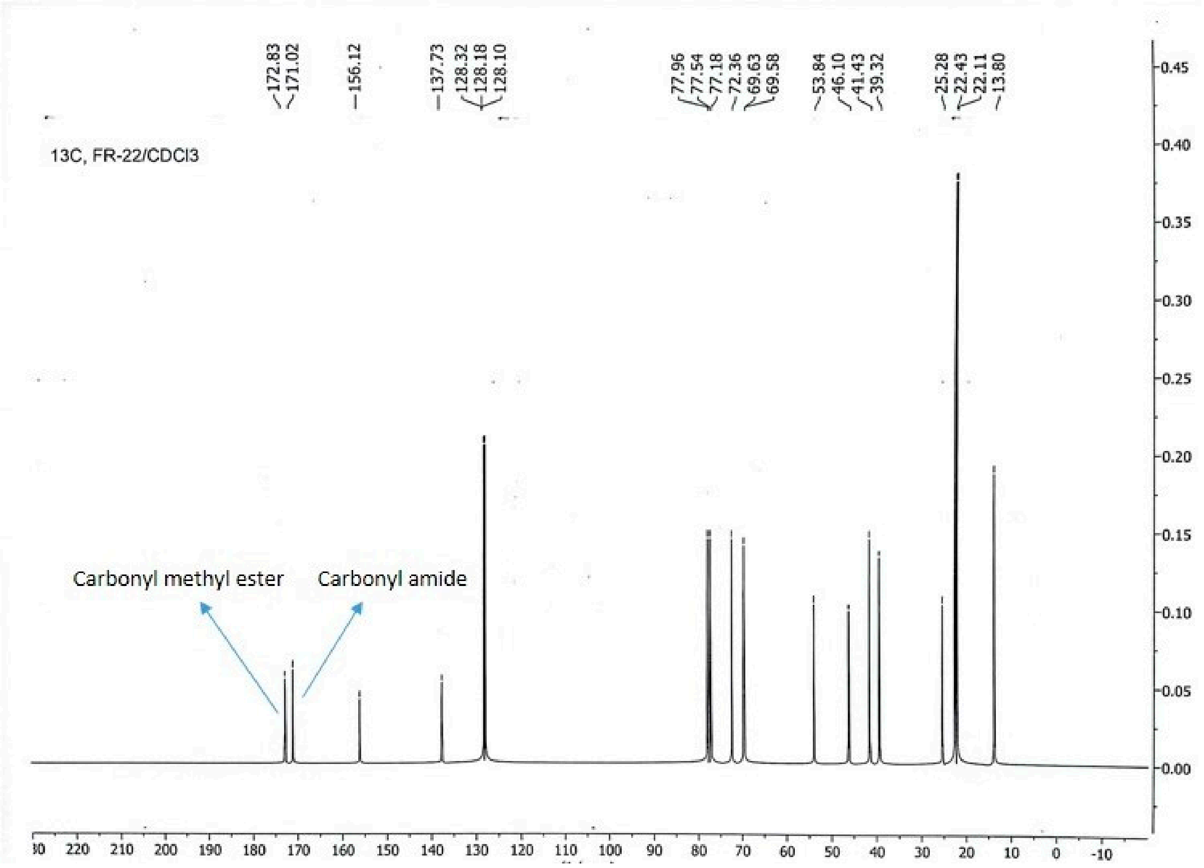

Figure S21. ^{13}**C NMR Spectrum of N-Boc-O-Bz-Serine-β-Leucine Dipeptide (16)**

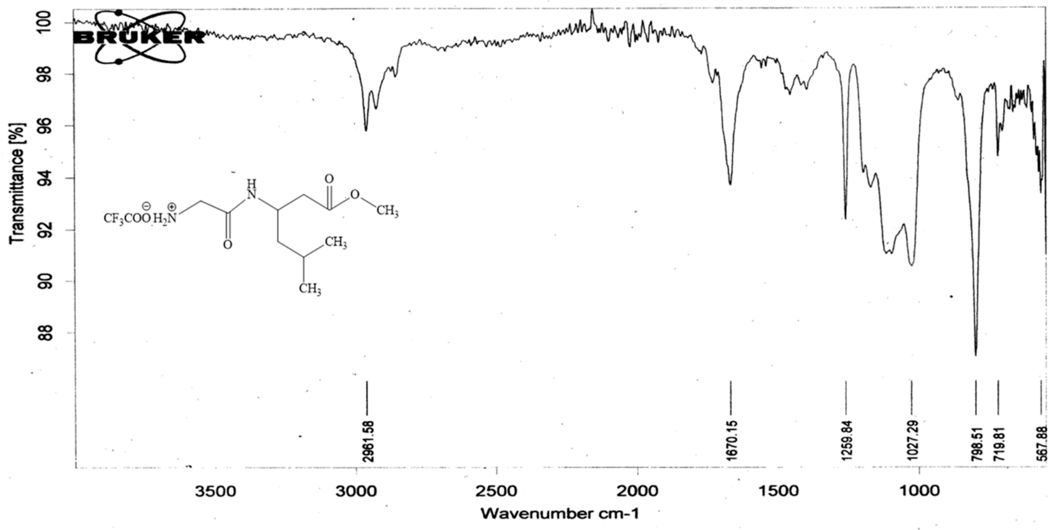

Figure S22. FTIR Spectrum of TFA-Gly-β-Leucine OCH_3 (16A)

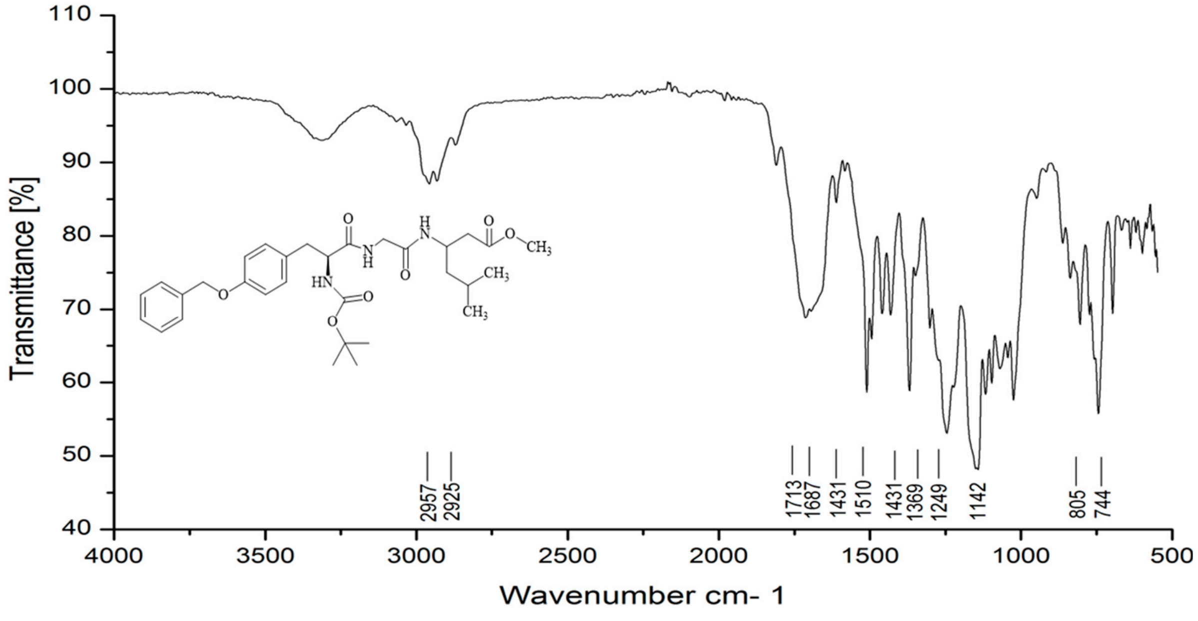

Figure S23. FTIR Spectrum of *N*-Boc-*O*-Bz-L-Tyrosine-L-Glycine-β-Leucine methyl Ester Tripeptide (17)

S

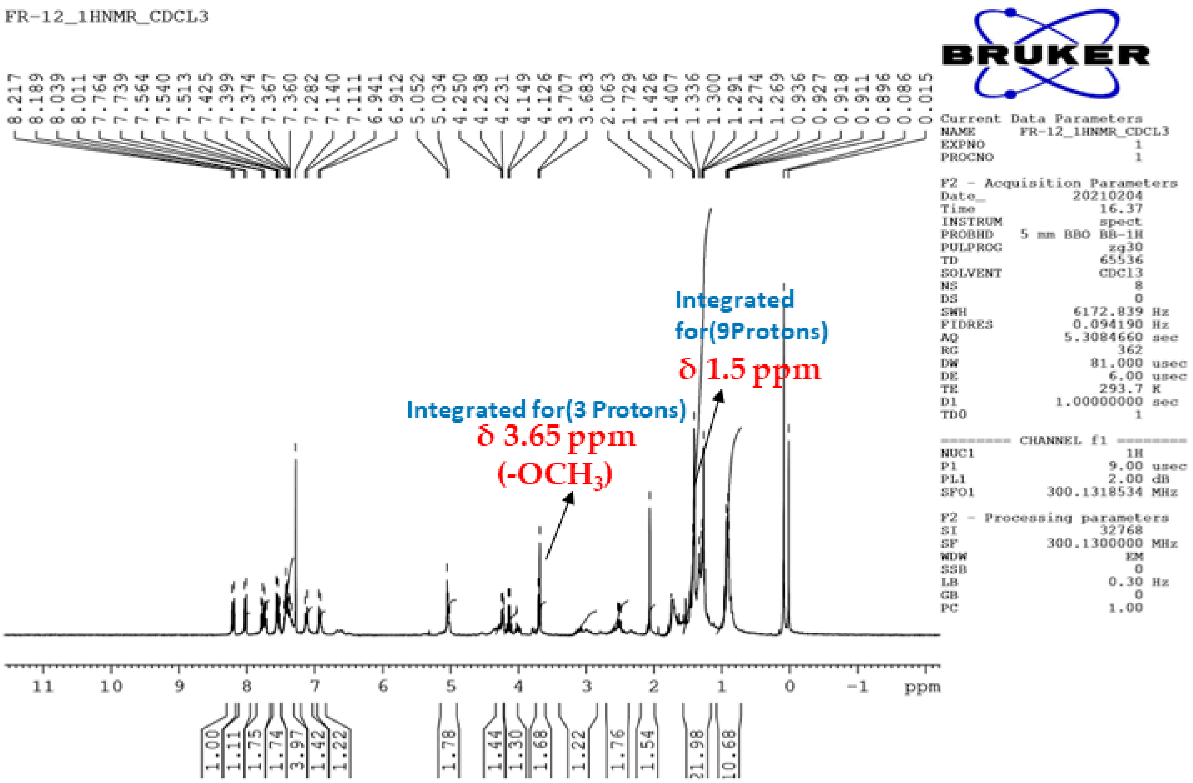

Figure S24. ^{1}HNMR Spectrum of α-Tyrosine-α-Glycine-β-Leucine Methyl Ester Tripeptide (17)

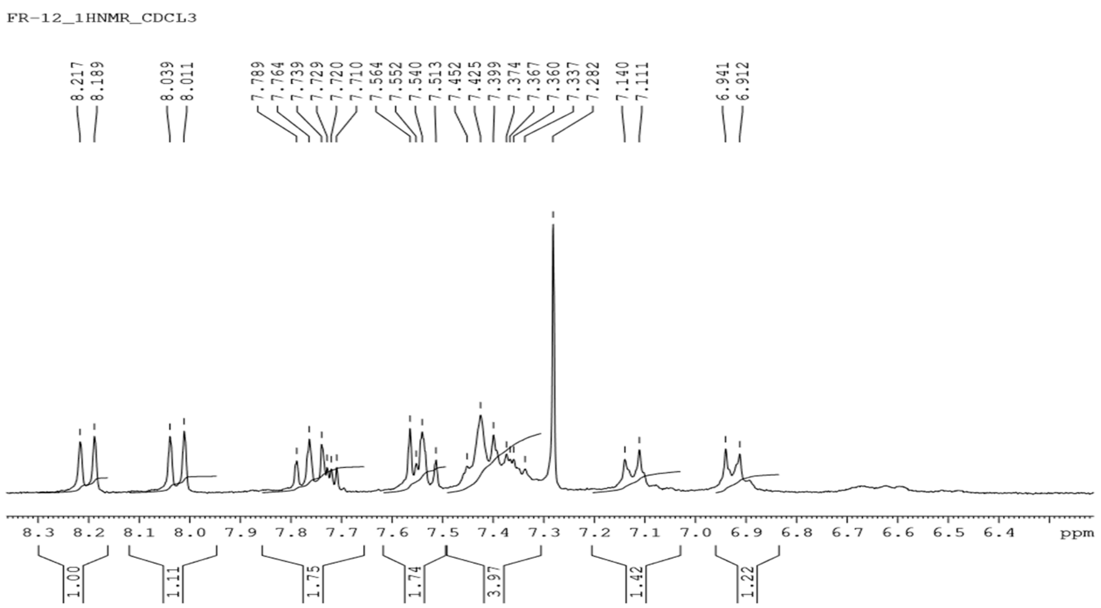

Figure S25. ^{1}**H NMR Spectrum (Extended) of α-Tyrosine-α-Glycine-β-Leucine methyl Ester (17)**

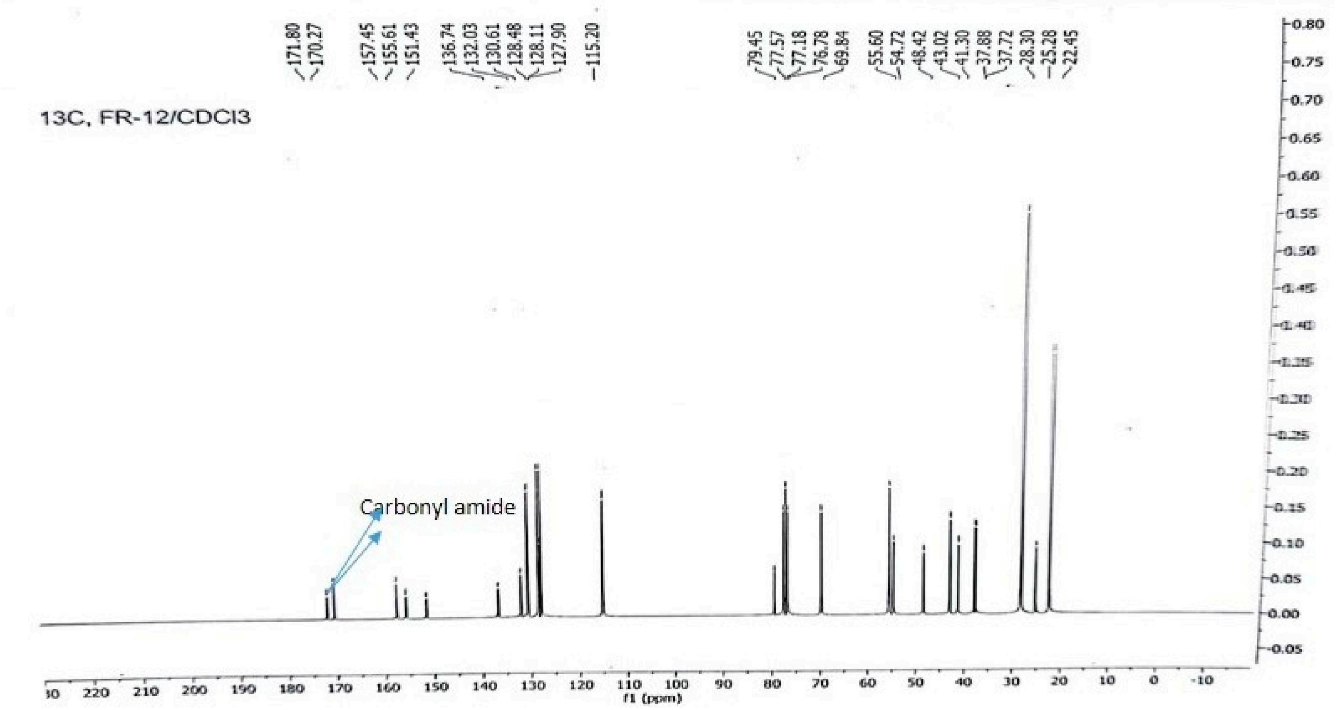

Figure S26. ^{13}C NMR Spectrum of α-Tyrosine-α-Glycine-β-Leucine methyl Ester (17)
